# Identification of circular RNAs in cardiac hypertrophy and cardiac fibrosis

**DOI:** 10.3389/fphar.2022.940768

**Published:** 2022-08-08

**Authors:** Yan Chen, Junteng Zhou, Zisong Wei, Yue Cheng, Geer Tian, Yue Quan, Qihang Kong, Wenchao Wu, Xiaojing Liu

**Affiliations:** ^1^ Laboratory of Cardiovascular Diseases, Regenerative Medicine Research Center, West China Hospital, Sichuan University, Chengdu, China; ^2^ Health Management Center, West China Hospital, Sichuan University, Chengdu, China; ^3^ Laboratory of Cardiovascular Diseases, West China Hospital, Sichuan University, Chengdu, China; ^4^ Department of Cardiology, West China Hospital, Sichuan University, Chengdu, China

**Keywords:** cardiac hypertrophy, cardiac fibrosis, inflammation, circRNA, ceRNA

## Abstract

Cardiac hypertrophy initially serves as an adaptive response to physiological and pathological stimuli. Sustained hypertrophy progress to pathological cardiac hypertrophy, cardiac fibrosis and ultimately lead to heart failure, one of the leading medical causes of mortality worldwide. Intervention of pathological cardiac hypertrophy can effectively reduce the occurrence of heart failure. Abundant factors, such as adrenergic, angiotensin, and endothelin (ET-1) receptors, have been shown to participate in the regulation of pathological cardiac hypertrophy. Recently, an increasing number of studies have indicated that circRNA and circRNA-miRNA–mRNA network regulation is indispensable for the posttranscriptional regulation of mRNA in cardiac hypertrophy. In our study, the morphological, cardiac function and pathological changes during cardiac hypertrophy were investigated. RNA sequencing identified 93 circRNAs that were differentially expressed in the TAC_2w group, and 55 circRNAs in the TAC_4w group compared with the sham group. Gene ontology (GO) and Kyoto Encyclopedia of Genes and Genomes (KEGG) pathway analyses identified several significant pathways, including hypertrophic cardiomyopathy, extracellular matrix (ECM)-receptor interaction and focal adhesion. Coexpression analyses were performed for differentially expressed circRNAs and differentially expressed mRNAs. Based on gene set enrichment analysis (GSEA), 8 circRNAs (mmu-Nfkb1_0001, mmu-Smad4_0007, mmu-Hecw2_0009, mmu-Itgbl1_0002, mmu-Lrrc2_0005, mmu-Cpeb3_0007, mmu-Ryr2_0040, and mmu-Rtn4_0001) involved in cardiac hypertrophy and cardiac fibrosis were identified. We validated some key circRNAs by qPCR. The crucial coexpression of circRNA–mRNA and its interaction with miRNA showed the possible mechanism of circRNAs in the process of cardiac dysfunction. Our results may provide promising targets for the treatment of pathological cardiac hypertrophy and fibrosis.

## Introduction

Cardiac hypertrophy is the compensatory response of myocardium caused by cardiac pressure overload, myocardial infarction, inflammation and other injury factors (Oka et al., 2014; Nakamura and Sadoshima, 2018). Cardiac fibrosis is caused by the accumulation of extracellular matrix proteins (collagens I, III, and proteases, among others) in myocardial tissue and in the microvasculature and the severity of fibrosis is associated with worse clinical outcomes. Sustained cardiac hypertrophy and fibrosis destroy the structure of the myocardium and develop into pathological cardiac hypertrophy which leading to myocardial disorganization, increased ventricular tissue stiffness, ventricular diastolic and systolic dysfunction, arrhythmias resulting from abnormal electrical conduction, and cardiac remodeling, which ultimately progresses to heart failure (Nakamura and Sadoshima, 2018; Nwabuo and Vasan, 2020; Ramachandra et al., 2021). Previous studies have partially revealed the pathological mechanisms of cardiac hypertrophy and fibrosis, such as oxidative stress and adrenergic (Burgoyne et al., 2012; Nomura et al., 2018). However, the etiology of cardiac hypertrophy is complex and multifactorial and remains difficult to fully elucidate. Due to the lack of specific treatments available for pathological cardiac hypertrophy, especially for cardiac fibrosis, a large proportion of patients still receive insufficient treatment. Thus, further exploration of the molecular mechanisms associated with pathological cardiac hypertrophy and identification of potential therapeutic targets are of considerable significance for delaying the progression of cardiac fibrosis, reversing maladaptive cardiac remodeling and improving clinical adverse prognosis.

Abundant RNAs have been reported to be extensively involved in cardiac hypertrophy. Commonly, mRNAs participate in the regulation of cardiac hypertrophy by directly coding functional proteins (e.g., angiotensin II, catecholamines and natriuretic peptides). According to the latest GENCODE version 19, although more than 90% of the mammalian genome is transcribed, only ∼2% of the transcripts encode proteins, and the vast majority are transcribed as noncoding RNAs (ncRNAs) (The ENCODE Project Consortium, 2012; Djebali et al., 2012). Emerging evidence has shown that ncRNAs, including microRNAs (miRNAs) and circRNAs, are critical regulators in the initiation and development of cardiac hypertrophy. miRNAs are a class of endogenous small RNA molecules (20–23 nucleotides in length) that negatively regulate their target gene expression post-transcriptionally (Valencia-Sanchez et al., 2006) and participate in various physiological processes of the cardiovascular system, such as proliferation, differentiation, oxidative stress, autophagy and apoptosis (Xu et al., 2004; Cheng et al., 2005; Ucar et al., 2012). Studies have confirmed that miR-1 (Karakikes et al., 2013; Seok et al., 2020), miR-133 (Carè et al., 2007), miR-29a (Roncarati et al., 2014), miR-21-3p (Yan et al., 2015), miR-155 (Seok et al., 2014), and miR-22 (Huang et al., 2013) are associated with cardiac hypertrophy. CircRNAs are a class of single-stranded endogenous RNAs with closed continuous back-spliced loop structures and without a free 3′ or 5′ end. CircRNAs are widely expressed in the heart and exhibit greater stability than linear RNAs (Siede et al., 2017; Tan et al., 2017; Chen, 2020). Recent studies have found that circRNAs are commonly dysregulated in cardiovascular diseases, including cardiac hypertrophy and fibrosis (Werfel et al., 2016; Tan et al., 2017). Multiple studies have revealed that circRNAs modulate mRNA expression in cardiovascular pathophysiology as miRNA sponges. CircNfix, a cardiac-specific circRNA, inhibited cardiomyocyte apoptosis and induced cardiac regeneration proliferation after myocardial infarction (MI) by sponging miR-214 (Huang et al., 2019). Another study identified the circMAP3K5/miR-22-3p/TET2 axis as a promising therapeutic target for the treatment of intimal hyperplasia (Zeng et al., 2021). Currently, only a few circRNAs have been reported to be explicitly related to pathological cardiac hypertrophy and cardiac fibrosis, such as circSlc8a1 (Lim et al., 2019) circRNA_000203 (Li et al., 2020) and circYap (Wu et al., 2021). Thus, it is worthwhile to identify the key circRNAs involved in the pathological cardiac hypertrophy and cardiac fibrosis process.

Consequently, in our study, we first constructed a mouse model of pressure overload-induced pathological cardiac hypertrophy at different stages (2 weeks and 4 weeks) by TAC operation. We focused on the phenotypic changes in different stages of cardiac hypertrophy and fibrosis. Furthermore, we systematically analyzed how circRNA and mRNA expression changes during cardiac hypertrophy. We aimed to gain insight into the gene expression pattern during pathological cardiac hypertrophy and identify some key circRNA-based ceRNA networks involved in pathological cardiac hypertrophy by bioinformatics. Our results may provide promising targets for the treatment of cardiac hypertrophy and fibrosis.

## Materials and methods

### The establishment of an animal model of cardiac hypertrophy

The SPF C57BL/6 mice (male, 6–8 weeks, 20–22 g) used in this study were purchased from the Experimental Animal Tech Co. of Weitonglihua (Beijing, China). All animal experiments were approved by the animal ethics committee of West China Hospital (Ethics number 2018162A).

Thoracic aortic coarctation (TAC) is a well-established method and is widely used to establish an animal model of pressure overload-induced cardiac hypertrophy. The TAC surgery was performed as described in our previous study (Zhou et al., 2020). Briefly, the mice were anesthetized with isoflurane (2.5% for induction, 1.0% for maintenance) and fixed on an autoadjusting heating pad, which maintained the body temperature at 37 °C. The mice were then intubated using PE 90 tubing, and the trachea was subjected to a small animal ventilator for mechanical ventilation. After the thoracotomy, the aortic arch was constricted with a 5–0 silk suture tied with a blunt 27-gauge needle. Then, we removed the needle before closing the chest. In the sham-operated group, a similar procedure was performed without ligation of the aorta.

The mice were randomly assigned to the following groups: sham, TAC_2w and sham, TAC_4w, with 6 mice per group. Two or 4 weeks after the operation, echocardiography was performed on all mice to assess cardiac function.

After echocardiography, the mice were euthanized, and the body weight was recorded. Then, the left tibia was isolated, and the tibia length was measured. A total of 20 mice were selected for histology, with five samples per group. At the end of the experiment, the left ventricles excised from the mice were frozen in liquid nitrogen and stored in a -80°C freezer for further analysis.

After successful establishment of TAC model, we performed whole transcriptome RNA-seq using 15 mice from Sham, TAC_2w and TAC_4w group (5 mice per group).

### Echocardiography

The Vevo^®^3100 imaging system (Fujifilm, Japan) fitted with a 35 MHz transducer was applied to evaluate the cardiac function of mice two- or 4-weeks following TAC operation. Briefly, mice were anesthetized with 2% isoflurane, and anesthesia was maintained with 0.5% isoflurane. Then, the mice were fixed on the monitoring table in the supine position. With the heart rate of the mice at 400–500 beats/min, B-mode and M-mode images were acquired. Using Vevo Lab 3.2.6 software, we analyzed these images and calculated the following indexes: left ventricular diastolic diameter (LVIDd), left ventricular systolic diameter (LVIDs), diastolic interventricular septal (IVSd) thickness, left ventricular posterior wall (LVPWd) thickness, left ventricular ejection fraction (EF) and fractional shortening (FS).

### Histological analysis

The hearts were excised and fixed in 4% paraformaldehyde. The fixed heart tissues were routinely dehydrated and paraffin-embedded. The wax blocks were cut into sections with a thickness of 4–5 μm. Subsequently, heart sections were stained with hematoxylin-eosin (HE, Servicebio, G1003, China) and Masson’s trichrome (Servicebio, G1006, China) to assess the size and morphologic alterations of the heart. FITC-conjugated wheat germ agglutinin (WGA, sigma, L4895, 1:500) staining was performed to demarcate cardiomyocyte boundaries, and DAPI (Servicebio, G1012, China) was used to label the nuclei. Staining images were captured using a microscope (Leica). The cardiomyocyte cross-sectional area and fibrosis area were analyzed by Image pro plus 6.0.

### RNA extraction, library preparation, and transcriptome sequencing

Total RNA was isolated from the left ventricular tissues of mice with TRIzolTM reagent (Invitrogen, United States). RNA integrity was assessed using the RNA Nano 6000 Assay Kit of the Bioanalyzer 2100 system (Agilent Technologies, CA, United States) prior to library preparation. The qualified RNA samples were treated with Ribo-Zero^TM^ Gold Kits (Epicenter Technologies, United States) to remove ribosomal RNA (rRNA). Sequencing libraries were generated using the NEBNext^®^ UltraTM RNA Library Prep Kit for Illumina^®^ (NEB, United States) following the manufacturer’s recommendations. The library preparations were sequenced on an Illumina HiSeq 4000 platform, and 125 bp/150 bp paired-end reads were generated.

### Gene quantification

Raw RNA-seq reads were trimmed with TrimGalore software (Trim, 2015) (http://www.bioinformatics.babraham.ac.uk/projects/trim_galore), including the removal of adaptor sequences and low-quality regions (Phred score ≥25). The clean reads were mapped to the GRCm38/mm10 mouse genome using the HISAT2 alignment tool (Kim et al., 2015) (http://daehwankimlab.github.io/hisat2). The mRNA level of each gene was quantified using Cuffdiff (Trapnell et al., 2013) (http://cole-trapnell-lab.github.io/cufflinks/cuffdiff). The raw RNA-seq data are available on SRA Bioproject accession PRJNA787574.

### circRNA identification and quantification

Based on the reference mouse genome (GRCm38/mm10), clean reads were subjected to CIRIquant software to identify circRNAs (Zhang et al., 2020). For convenience, the GRCm38 GTF file was used to convert the specified gene IDs to gene symbols. (http://ftp.ensembl.org/pub/release102/gtf/mus_musculus/Mus_musculus.GRCm38.102.gtf.gz). Briefly, the unaligned sequences were applied to CIRI2 (Gao et al., 2018) to identify back-spliced junction sites (BSJs) and obtain potential circRNAs. To accurately quantify the expression level of circRNA, two full-length sequences of the BSJ region were connected to construct a pseudo circRNA reference sequence. Then, the potential circRNA was realigned to the pseudosequence. Combining the results with genome and pseudo circRNA sequences, the junction rate of each circRNA can be determined by calculating the ratio of the circular junction reads. The identified circRNAs were mapped to the circAltas 2.0 (Wu et al., 2020) database to obtain annotation information. A total of 50319 circRNAs were initially identified in all samples; however, to reduce false positives of circRNA identification, only those circRNAs with two junction reads in at least two samples were retained. Finally, 6643 circRNAs with high confidence were used for all subsequent analyses.

### Differential expression analysis and trend analysis

Differential expression analysis of circRNA was performed using the DE pipeline in CIRIquant using edgeR (Robinson et al., 2010) according to the criteria of *p* value < 0.01 and |log2 (fold change) | > 1. The statistically significant DE mRNAs were obtained by an adjusted *p* value < 0.05 and |log2 (fold change) | > 1 using edgeR software. To obtain detailed information on the progression in different periods after TAC, the DE circRNAs were clustered into eight profiles based on gene expression patterns using STEM software (Ernst and Bar-Joseph, 2006). DE circRNAs clustered to the same module were considered to have similar expression patterns at different stages of disease progression.

### Coexpression network analysis

The Pearson’s correlation coefficient and *p* value of each DE circRNA and mRNA were calculated by Pearson correlation analysis. To identify circRNA-mRNA pairs, the criterion for inclusion was a correlation coefficient of >0.85 and a *p* value <0.01. Then, the circRNA-mRNA were collected to construct a coexpression network and visualized using Cytoscape software (http://www.cytoscape.org/).

### Functional enrichment analysis

Analysis of the GO, KEGG and Reactome pathways in which circRNA-related coexpressed mRNAs and circRNA-related host genes were involved was performed using DAVID (https://david.ncifcrf.gov) and Metascape (www.metascape.org). The terms with enrichment *p* value < 0.05 were considered significant.

### ceRNA network construction

Sequences of the DE circRNAs were downloaded from the circAtlas 2.0 database. Then, RNAhybrid (Krüger and Rehmsmeier, 2006) and miRanda (Betel et al., 2008) were used to predict target miRNAs of selected circRNAs. The miRNAs predicted by both tools were retained for further analysis. Next, we used TargetScan (http://www.targetscan.org/), miRDB (http://mirdb.org/miRDB), miRTarBase (http://mirtarbase.mbc.nctu.edu.tw) and miRWalk (http://mirwalk.umm.uni-heidelberg.de/) to identify miRNA-targeted mRNAs. The predicted miRNA-targeted mRNAs that existed in at least three databases were included. Then, these genes that intersected with the DE mRNAs were selected for interaction network construction. Finally, we integrated the circRNA–mRNA coexpression and circRNA, miRNA and mRNA regulatory relationships to construct a ceRNA network and visualized it using Cytoscape software (http://www.cytoscape.org/).

### Cell isolation, culture, and treatment

Neonatal mice cardiomyocytes (NMCMs) and neonatal mice cardiac fibroblasts (NMCFs) were isolated from the hearts of neonatal C57BL mice (0–3 days after birth) according to the protocol reported previously (Sreejit et al., 2008; Xin et al., 2019). Then, the NMCMs and NMCFs were grown in a culture plate with DMEM (Gibico, C11995500BT, United States) containing 10% fetal bovine serum (FBS, Gibico, 10099141C, United States) with 100 U/ml streptomycin and penicillin (Hyclone, SV30010, United States).

SiRNA targeting mmu-Hecw2_0009 (70 nM, Ribo, Guangzhou, China) was transfected into NMCMs by lipofectamine RNAiMax (Invitrogen, 13778150,United States) for 24 h and then treated with Phenylephrine (PE, 200 μM, Cat. #HY-B0471, MCE, United States) for further 24 h. The sequences of mmu-Hecw2_0009 siRNA is as follows: CAT​CAG​GGC​AAG​ACT​GGG​A.

### Quantitative real-time PCR

To validate circRNA expression, we designed divergent primers and performed qRT–PCR. In brief, total RNA was isolated from mouse hearts using TRIzol^TM^ reagent (Invitrogen, 15596018, United States). Then, reverse transcription was accomplished using a reverse transcription kit (TOYOBO, FSQ-101, Japan). Next, qRT–PCR was performed to detect gene expression using the ChamQ Universal SYBR qPCR Master Mix Kit (Vazyme, Q711-03, China) on a CFX96 detection system (Bio–Rad, United States). The relative expression of circRNA was calculated based on the cycle threshold values according to the 2^−ΔΔCt^ method. β-actin was used as a reference gene for normalization. The primers used are listed in Supplementary Table S1.

### Immunofluorescence

To assess the cell size of NMCMs, the NMCMs were fixed in 4% paraformaldehyde for 20 min and permeabilized with 0.25% Triton X-100 for 10 min, followed by incubated with α-actinin primary antibody (Sigma Aldrich, A7811, 1:200, United States) overnight at 4°C. After washing, samples were incubated for 2 h at room temperature with fluorochrome-conjugated secondary antibodies (Bioss, bs-0296G-AF594, 1:400, China). Nuclei are stained with DAPI (Sigma Aldrich, D9542, 1:2000) for 5 min. Immunofluorescent micrographs were obtained using a laser-scanning confocal microscope (Leica Stellaris 5, Germany). The size of cardiomyocytes was analyzed by Image pro plus 6.0.

### Statistical analysis

Data are presented as the mean ± standard error of the mean (SEM). Unpaired Student’s t test (two-tailed) was used for comparisons between groups or one-way ANOVA test was performed to analyze multiple groups followed by Bonferroni post hoc tests. All data were analyzed using GraphPad Prism 7. *p* value < 0.05 was considered significant.

## Results

### Evaluation of mouse cardiac hypertrophy models

**FIGURE 1:**
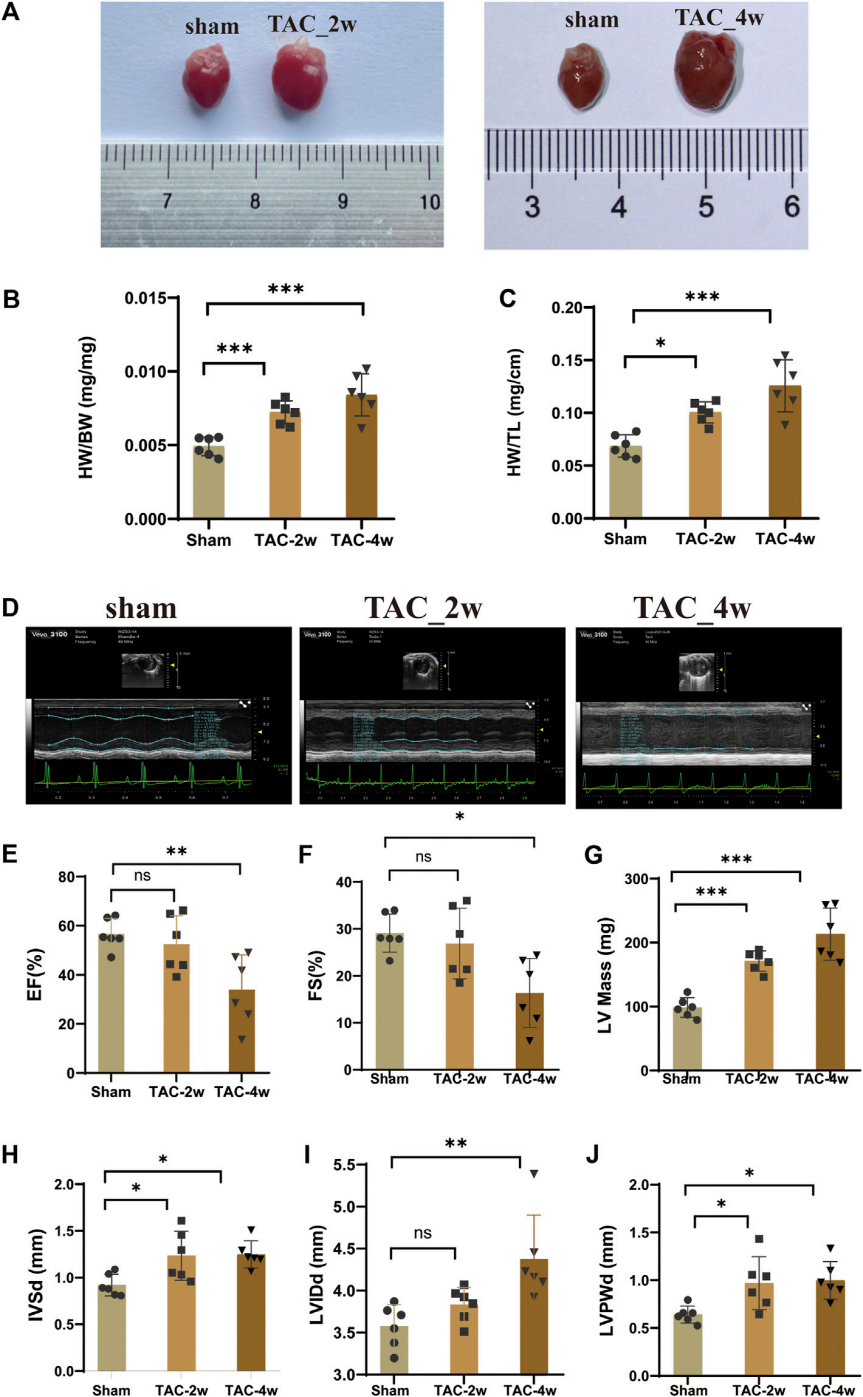
Evaluation of mouse cardiac hypertrophy models. **(A)** The heart appearance of sham, TAC_2w and TAC_4w group. **(B)**The heart weight/body weight (HW/BW) significantly increased in TAC2w and TAC4w compared to the sham group. **(C)**The heart mass/tibia length (HW/TL) significantly increased in TAC2w and TAC4w compared to the sham group. **(D)** Representative image of an echocardiographic detection of the sham, TAC_2w and TAC_4w. **(E–J)** Cardiac function indicators (EF, FS, LV mass, LVIDd, LVPWd) measured by echocardiography in sham, TAC_2w and TAC_4w.

**FIGURE 2:**
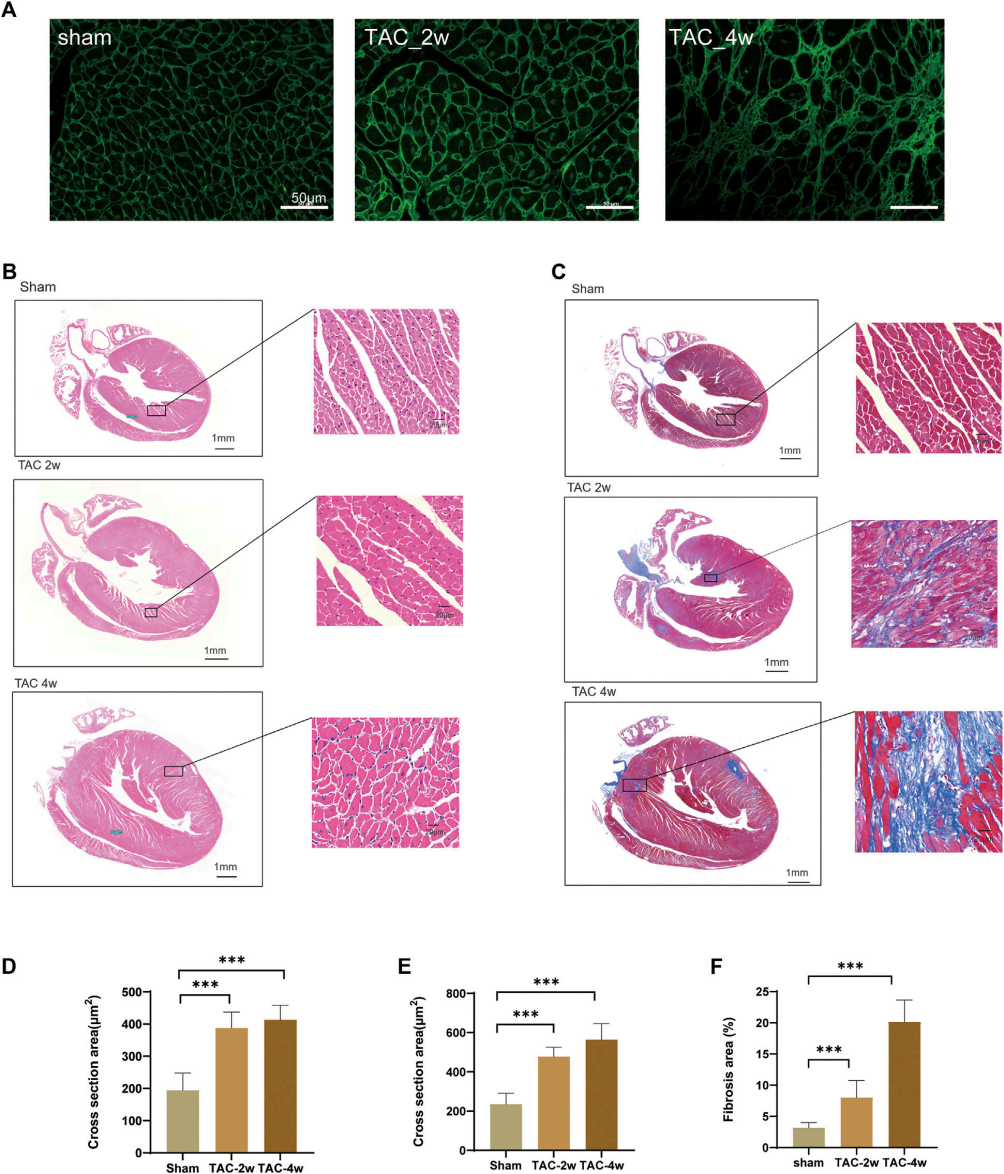
Evaluation of mouse cardiac hypertrophy models. **(A,D)** Representative WGA images and the statistical result of cross section area of mouse hearts. **(B,E)** Representative HE images and the statistical result of cross section area of mouse hearts. **(C,F)** Representative Masson trichrome staining images and the statistical result of fibrosis area mouse hearts. Results are presented as means ± standard error of the mean; ∗ indicates *p* < 0.05, ∗∗*p* < 0.01, ∗∗∗*p* < 0.001. *n* = 6.

To observe the dynamic changes in cardiac anatomy and function in different stages of cardiac hypertrophy, we first established a pressure overload-induced cardiac hypertrophy mouse model at 2 and 4 weeks after TAC surgery. Echocardiography was performed at 2 w and 4 w after sham operation and TAC, respectively. We found that the hearts of mice in the TAC_2w group exhibited compensatory hypertrophy, and the cardiac function was still within the normal range. Compared with the sham group, the LV mass, IVSd and LVPW of the TAC_2w group were significantly increased (Figures 1G,H,J), but there was no significant difference in EF, FS, or LVIDd (Figures 1E,F,I). The mouse hearts in the TAC_2w group were larger than those in the sham group, with the heart weight/body weight (HW/BW) and the heart mass/tibia length (HW/TL) significantly increased (Figures 1B,C). The results of HE staining and WGA staining showed that the cross-sectional area of the myocardium of the mice in the TAC_2w group increased (Figures 2A,B,D,E), and Masson staining showed that the myocardium of the TAC_2w group began to have slight collagen deposition (Figures 1C,F). Compared with the sham group, the cardiac function of the TAC_4w mice was significantly worsened after the TAC operation. The TAC_4w group mainly manifested as increased EF, FS, LV mass, LVIDd, LVPWd, cardiac shape, cross-sectional myocardial, myocardial collagen deposition and cardiac fibrosis (Figures 1A–J, Figures 2A–F).

### Identification of differentially expressed circRNAs and mRNAs (DE mRNAs) during cardiac hypertrophy

**FIGURE 3:**
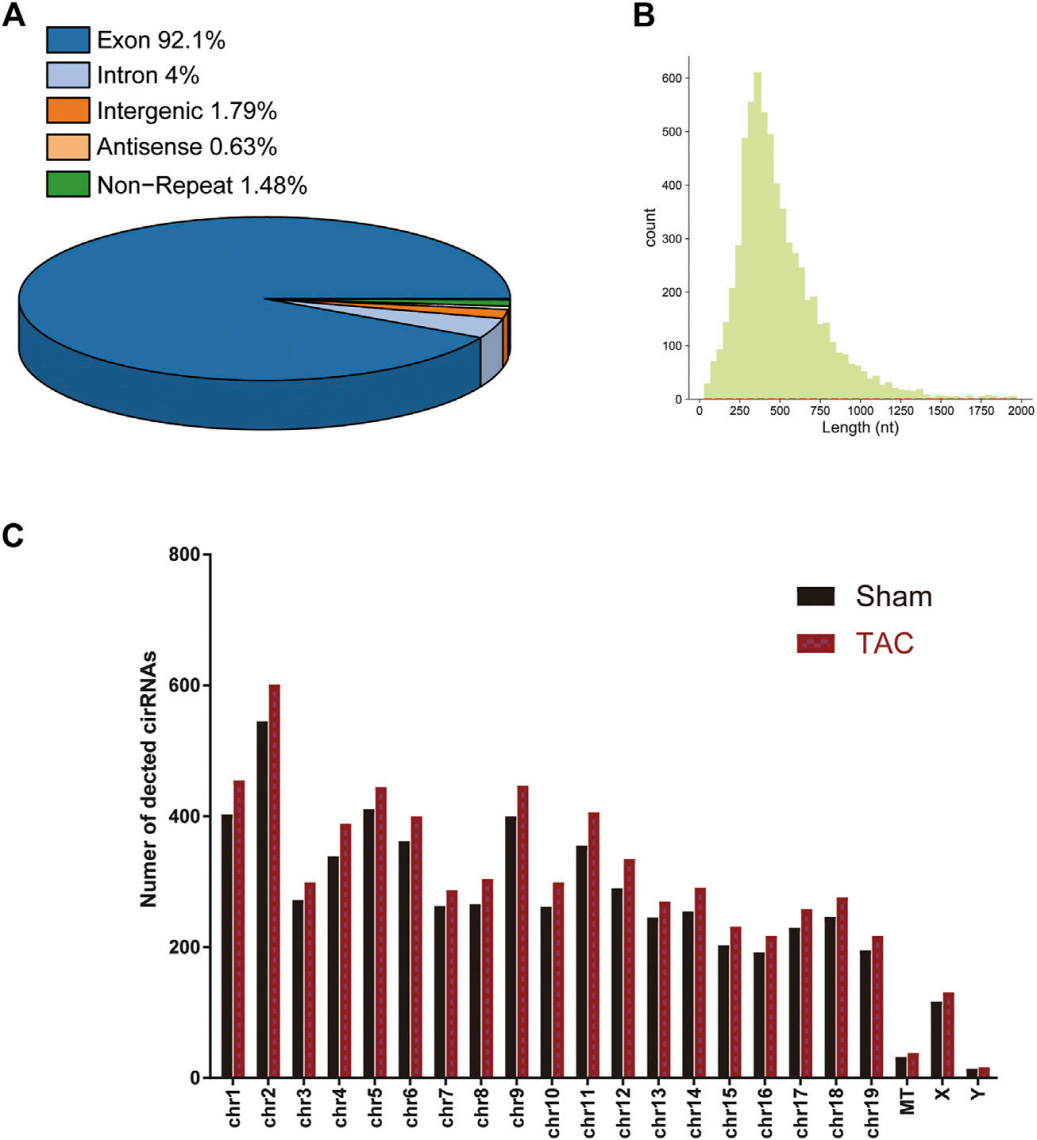
Distribution profiles of circRNAs. **(A)** Class distribution of circRNAs. **(B)** Length distribution of circRNAs. **(C)** Chromosomal distribution of all circRNAs.

A total of 15 samples from three groups (sham, TAC_2w, TAC_4w) were subjected to whole-transcriptome RNA sequencing. We identified a total of 6643 circRNAs in the three groups (Supplementary Table S2). Based on the locations in their host genes, the circRNAs were classified into four categories: 92.1% exonic, 4% intronic, 1.79% intergenic and 0.63% antisense (Figure 3A). The majority of the circRNAs in the three groups ranged in length from 31 to 1970 bp, and 6643 circRNAs were distributed across all chromosomes (Figures 3B,C).

**FIGURE 4:**
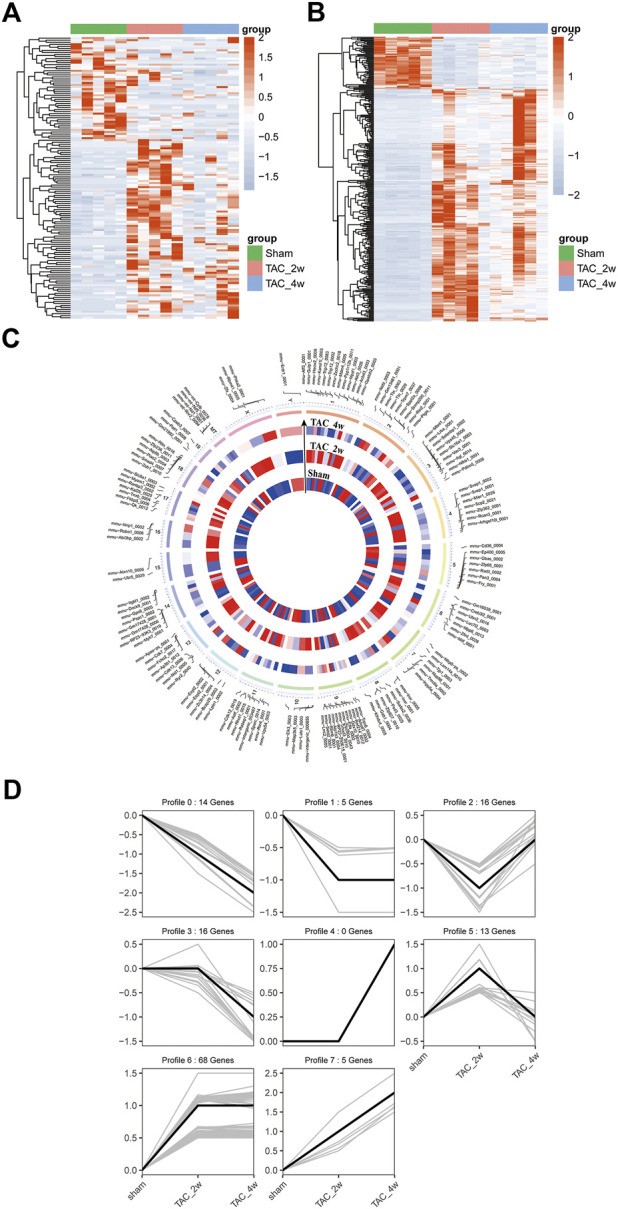
Identification of differentially expressed circRNAs (DE circRNAs) and mRNAs (DE mRNAs) during cardiac hypertrophy. **(A)** The heat map of DE circRNAs in sham, TAC_2w and TAC_4w. **(B)** The heat map of DE mRNAs in sham, TAC_2w and TAC_4w. **(C)** Chromosomal distribution of differentially expressed circRNAs in sham, TAC_2w and TAC_4w. **(D)** 7 expression trend profiles of DE circRNAs in sham, TAC_2w and TAC_4w.

To explore the alteration of circRNA in cardiac hypertrophy and cardiac fibrosis, we performed differential expression analysis on the circular RNAs of sham and TAC. Cluster analysis showed tight clustering of each experimental condition (Figure 4A). Compared with the sham group, 73 circRNAs were upregulated and 20 circRNAs downregulated in the TAC_2w group, and 18 circRNAs upregulated and 37 downregulated in the TAC_4w group (Figure 4C). The Supplementary Tables S3, S4 showed the basic information of top 10 upregulated and downregulated circRNAs in the hearts of TAC_2w vs. sham group and TAC_4w vs. sham group (Supplementary Tables S3, S4).

Based on the DE circRNA expression, we defined 7 expression trend profiles. These trend profiles clearly classified the expression alterations of these differential circRNAs in the sham, TAC_2w and TAC_4w groups (Figure 4D). Here, we focus specifically on profiles 0, 1 and 6 due to the biological significance of the expression pattern.

Likewise, we performed mRNA cluster analysis. The TAC group was significantly separated from the sham group in the heatmaps (Figure 4B). According to the screening criteria, a total of 3639 differentially expressed mRNAs were obtained, of which 1567 were upregulated and 970 downregulated in the TAC_2w group and 804 upregulated and 298 downregulated in the TAC_4w group.

### Functional enrichment analysis of the parent genes of circRNAs

**FIGURE 5:**
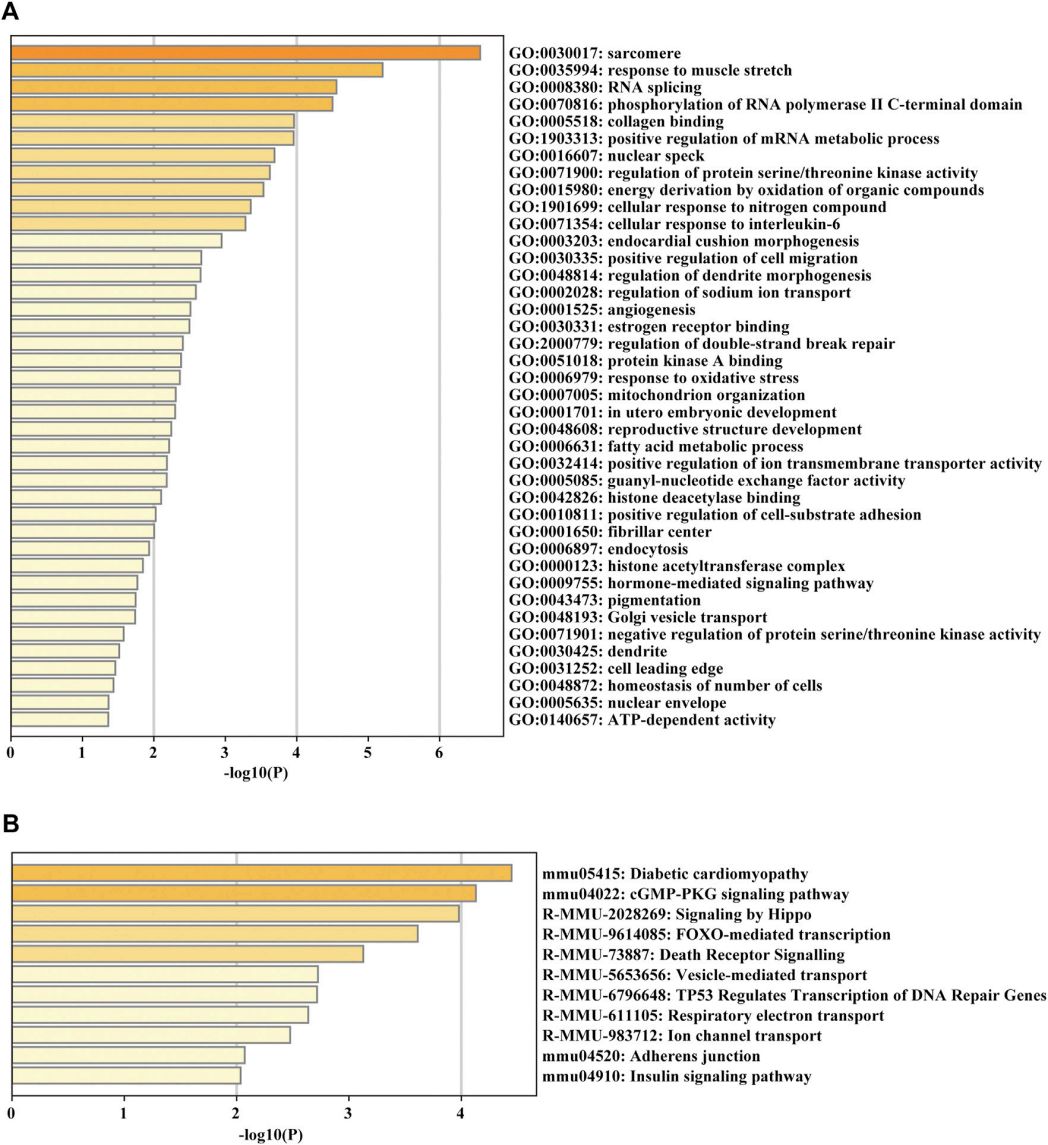
GO enrichment and KEGG pathway analyses of the parent genes of circRNAs. **(A)** GO analysis of the DE parent genes of circRNAs. **(B)** KEGG pathway enrichment analysis of the DE parent genes of circRNAs.

For glean insight into the potential biological function of the circRNAs, we annotated the parent genes of circRNAs by GO, KEGG and Reactome pathway enrichment analysis. The biological process results indicated that they were mainly enriched in GO:0030017 sarcomere, GO:0035994 response to muscle stretch, GO:0005518 collagen binding and GO:0006979 response to oxidative stress (Figure 5A). KEGG and Reactome pathway analysis showed that the parent genes of circRNAs were mostly enriched in several pathways related to cardiac hypertrophy and fibrosis formation, such as diabetic cardiomyopathy and adherens junction (Figure 5B).

### Coexpression of circRNA and mRNA

**FIGURE 6:**
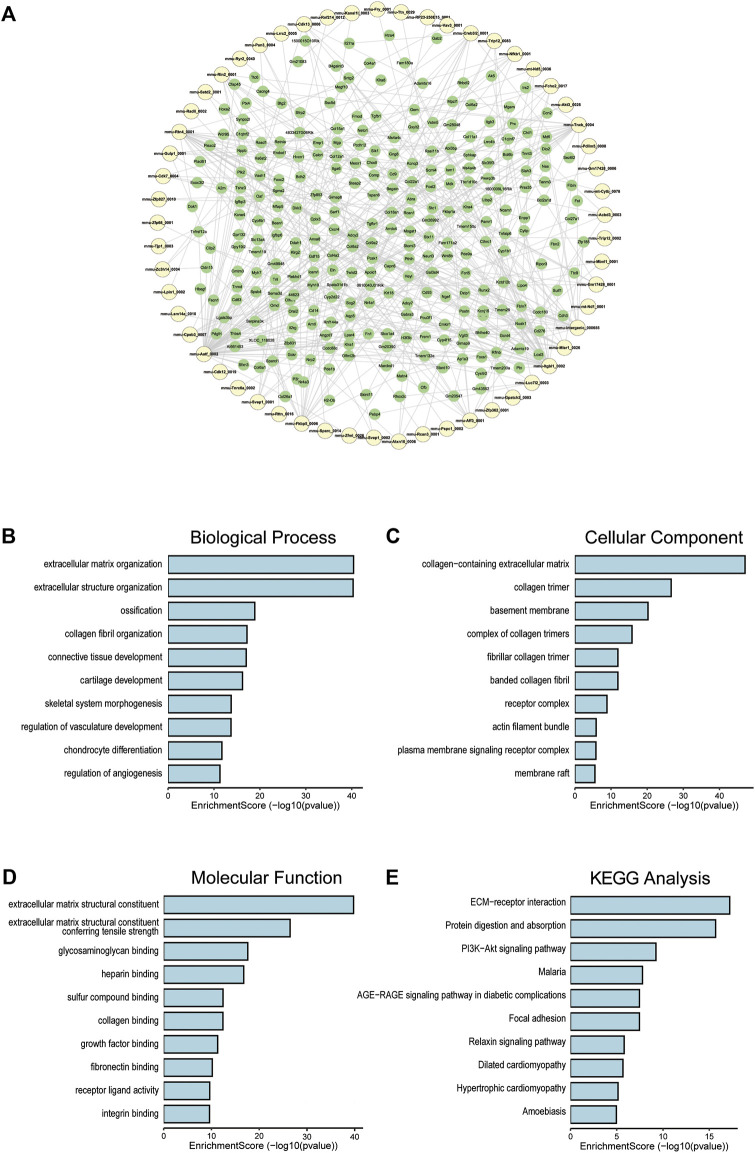
Co-expression of DE circRNA and DE mRNA. **(A)** Co-expression network of circRNA and mRNA, circRNA in yellow and mRNA in green. **(B)** The top 10 terms related to biological processes of DE mRNAs (coexpressed with DE circRNAs). **(C)** The top 10 terms related to cellular component of DE mRNAs (coexpressed with DE circRNAs). **(D)** The top 10 terms related to molecular function of DE mRNAs (coexpressed with DE circRNAs). **(E)** The top 10 enriched KEGG pathways.

To clarify the coexpression relationship between DE circRNAs and DE mRNAs, we identified 335 coexpression pairs (*p* < 0.01) and constructed a coexpression network for DE circRNAs and DE mRNAs (Figure 6A). Then, we performed functional enrichment analysis on these coexpressed mRNAs. Figures 6B–D and Supplementary Figures 1–3 showed DE mRNAs related to biological processes (BP), cellular component (CC) and molecular function (MF). The top 10 GO terms related to BP are shown in Table 1. The results of GO analysis revealed that these mRNAs (coexpressed with DE circRNAs) were mainly associated with collagen/fibrosis formation, such as extracellular matrix organization and collagen fibril organization. The top 10 enriched KEGG pathways are shown in Figure 6E and Table 2. Specifically, KEGG analysis determined that the top pathways included extracellular matrix (ECM)-receptor interactions, focal adhesion, the PI3K-AKT signaling pathway and hypertrophic cardiomyopathy. In summary, the alteration of these pathways is closely related to cardiac hypertrophy, the inflammatory response and cardiac fibrosis (Figure 6E and Supplementary Figures S4).

**TABLE 1:** Top 10 terms with the largest significant differences in the GO biological process analysis of the mRNAs co-expressed with circRNAs.

ID	Term	Count	GeneRatio	BgRatio	Pvalue	Candidate gene
GO:0030198	extracellular matrix organization	70	70/717	302/23328	3.99E-41	Col9a2/Col11a1/Comp/Col8a2/Col13a1/Col22a1/Abi3bp/Col5a3/Myo1e/Tgfbr1/Itgb3/Adamts1/Cyp1b1/Eln/Col27a1/Loxl3/Mmp14/Col1a1/Sulf1/Aebp1/Col14a1/Col5a2/Col5a1/Postn/Col1a2/Loxl1/Scx/Adamts2/Olfml2b/Col16a1/Sh3pxd2b/Mfap4/Col8a1/Tgfb1/Ccn2/Slc2a10/Ccn1/Col15a1/Col4a1/Col4a2/Crispld2/Tgfb2/Foxc2/Bcl3/Tnfrsf11b/Fscn1/Col18a1/Lox/Fn1/Adamts16/Sfrp2/Angptl7/Mmp2/Antxr1/Nid1/Tnfrsf1b/Lum/Wt1/Adamts9/Lamb3/Kif9/Lgals3/Adtrp/Col4a4/Papln/Acan/Npnt/Sox9/Adamts19/Col4a3
GO:0043062	extracellular structure organization	70	70/717	303/23328	5.04E-41	Col9a2/Col11a1/Comp/Col8a2/Col13a1/Col22a1/Abi3bp/Col5a3/Myo1e/Tgfbr1/Itgb3/Adamts1/Cyp1b1/Eln/Col27a1/Loxl3/Mmp14/Col1a1/Sulf1/Aebp1/Col14a1/Col5a2/Col5a1/Postn/Col1a2/Loxl1/Scx/Adamts2/Olfml2b/Col16a1/Sh3pxd2b/Mfap4/Col8a1/Tgfb1/Ccn2/Slc2a10/Ccn1/Col15a1/Col4a1/Col4a2/Crispld2/Tgfb2/Foxc2/Bcl3/Tnfrsf11b/Fscn1/Col18a1/Lox/Fn1/Adamts16/Sfrp2/Angptl7/Mmp2/Antxr1/Nid1/Tnfrsf1b/Lum/Wt1/Adamts9/Lamb3/Kif9/Lgals3/Adtrp/Col4a4/Papln/Acan/Npnt/Sox9/Adamts19/Col4a3
GO:0001503	ossification	53	53/717	394/23328	1.34E-19	Dhh/Twist2/Atp6v0a4/Mdk/Shox2/Comp/Col13a1/Ptn/Hoxa2/Stc1/Igfbp3/Fbn2/Egr2/Pthlh/Mmp14/Col1a1/Runx1/Col1a2/Scx/Igsf10/Enpp1/Runx2/Sh3pxd2b/Sgms2/Tmem119/Bcl2/Tgfb1/Ccn2/Ccn1/Cd276/Foxc2/Ccn4/Lox/Cthrc1/Fgf23/Rflnb/Aspn/Sfrp2/Thbs3/Mgp/Sfrp1/Mmp2/Omd/Junb/Cebpd/Cnmd/Npnt/H3f3b/Sox9/Clec11a/Acp5/Clic1/Bmp7
GO:0030199	collagen fibril organization	21	21/717	56/23328	6.39E-18	Col11a1/Comp/Col13a1/Tgfbr1/Cyp1b1/Loxl3/Col1a1/Aebp1/Col14a1/Col5a2/Col5a1/Col1a2/Loxl1/Scx/Adamts2/Tgfb2/Foxc2/Lox/Sfrp2/Lum/Acan
GO:0061448	connective tissue development	42	42/717	276/23328	9.55E-18	Prrx2/Mdk/Shox2/Col11a1/Comp/Tgfbr1/Stc1/Itgb3/Arid5a/Creb3l2/Thbs1/Col27a1/Pthlh/Col1a1/Sulf1/Runx1/Col5a1/Frzb/Scx/Runx2/Sh3pxd2b/Tgfb1/Ccn2/Ccn1/Chst11/Tgfb2/Foxc2/Ccn4/Lox/Rflnb/Sfrp2/Thbs3/Ctsk/Mgp/Matn3/Wt1/Efemp1/Cnmd/Acan/Sox9/Egr1/Bmp7
GO:0051216	cartilage development	35	35/717	200/23328	6.14E-17	Prrx2/Mdk/Shox2/Col11a1/Comp/Tgfbr1/Stc1/Arid5a/Creb3l2/Thbs1/Col27a1/Pthlh/Col1a1/Sulf1/Runx1/Frzb/Scx/Runx2/Tgfb1/Ccn2/Ccn1/Chst11/Tgfb2/Ccn4/Rflnb/Sfrp2/Thbs3/Ctsk/Mgp/Matn3/Efemp1/Cnmd/Acan/Sox9/Bmp7
GO:0048705	skeletal system morphogenesis	36	36/717	253/23328	1.82E-14	Twist2/Prrx2/Wnt9b/Shox2/Col11a1/Comp/Mdfi/Col13a1/Hoxa2/Tgfbr1/Stc1/Fbn2/Thbs1/Col27a1/Pthlh/Frem1/Mmp14/Col1a1/Tgfb3/Scx/Runx2/Sh3pxd2b/Tmem119/Ccn2/Chst11/Tgfb2/Foxc2/Rflnb/Sfrp2/Thbs3/Sfrp1/Mmp2/Acan/Sox9/Acp5/Bmp7
GO:1901342	regulation of vasculature development	43	43/717	356/23328	1.97E-14	Mdk/Tnmd/Ptn/Plk2/Itgb3/Adamts1/Cyp1b1/Thbs1/Chil1/Serpinf1/Sulf1/Ptgis/Runx1/Ddah1/Sparc/Tnfrsf12a/Col4a2/Tgfb2/Foxc2/Cd40/Ccl11/Sfrp2/Angptl7/Ism1/Ccl5/Cd34/Cxcr4/Wt1/Adamts9/Sphk1/Kit/Aplnr/Cnmd/Enpp2/Lgals3/Vash1/Tbxa2r/Cysltr2/Egr1/Adgra2/Col4a3/Bmp7/Cth
GO:0002062	chondrocyte differentiation	22	22/717	109/23328	1.92E-12	Mdk/Shox2/Col11a1/Comp/Tgfbr1/Arid5a/Creb3l2/Col27a1/Pthlh/Sulf1/Runx1/Scx/Runx2/Tgfb1/Ccn2/Chst11/Ccn4/Rflnb/Sfrp2/Efemp1/Acan/Sox9
GO:0045765	regulation of angiogenesis	37	37/717	320/23328	2.37E-09	Mdk/Tnmd/Ptn/Plk2/Itgb3/Adamts1/Cyp1b1/Thbs1/Chil1/Serpinf1/Sulf1/Ptgis/Runx1/Ddah1/Sparc/Tnfrsf12a/Col4a2/Tgfb2/Foxc2/Cd40/Ccl11/Sfrp2/Ism1/Ccl5/Cd34/Cxcr4/Adamts9/Sphk1/Aplnr/Cnmd/Enpp2/Lgals3/Vash1/Tbxa2r/Cysltr2/Adgra2/Col4a3

**TABLE 2:** The top10 biological pathways with the largest significant differences in the KEGG pathway analysis of DE mRNAs.

ID	Term	Count	GeneRatio	BgRatio	Pvalue	Candidate gene
mmu04512	ECM-receptor interaction	27	27/339	88/8949	6.14E-18	Itga6/Itgb4/Col9a2/Comp/Itgb3/Thbs4/Itgb6/Thbs1/Frem1/Fras1/Col1a1/Col1a2/Itga11/Col6a1/Col6a2/Col4a1/Col4a2/Itga9/Sdc1/Fn1/Thbs3/Sv2c/Lamb3/Col6a6/Col4a4/Npnt/Col4a3
mmu04974	Protein digestion and absorption	28	28/339	108/8949	2.03E-16	Col26a1/Col9a2/Col11a1/Col8a2/Col13a1/Col22a1/Col5a3/Eln/Col27a1/Col1a1/Col14a1/Col7a1/Col5a2/Col5a1/Col1a2/Col12a1/Col16a1/Col8a1/Col6a1/Col6a2/Col15a1/Col4a1/Col4a2/Col18a1/Col6a6/Col4a4/Slc6a19/Col4a3
mmu04151	PI3K-Akt signaling pathway	40	40/339	359/8949	5.89E-10	Itga6/Itgb4/Gng12/Col9a2/Comp/Lpar4/Itgb3/Thbs4/Itgb6/Creb3l2/Thbs1/Col1a1/Col1a2/Itga11/Bcl2/Osm/Col6a1/Col6a2/Ereg/Col4a1/Col4a2/Itga9/F2r/Gngt2/Fn1/Fgf23/Thbs3/Gng8/Ppp2r2c/Tlr2/Nr4a1/Cdkn1a/Kit/Lamb3/Col6a6/Col4a4/Il2rb/Il2rg/Csf3r/Col4a3
mmu05144	Malaria	14	14/339	57/8949	1.68E-08	Icam1/Klra4/Comp/Thbs4/Thbs1/Tgfb3/Lrp1/Tgfb1/Tgfb2/Cd40/Sdc1/Thbs3/Tlr2/Ccl12
mmu04933	AGE-RAGE signaling pathway in diabetic complications	18	18/339	101/8949	3.49E-08	Icam1/Nox4/Tgfbr1/Col1a1/Tgfb3/Col1a2/Bcl2/Tgfb1/Col4a1/Col4a2/Tgfb2/Fn1/Mmp2/Col4a4/Ccl12/Egr1/Casp3/Col4a3
mmu04510	Focal adhesion	26	26/339	201/8949	3.53E-08	Itga6/Itgb4/Col9a2/Comp/Itgb3/Thbs4/Itgb6/Thbs1/Col1a1/Col1a2/Pak3/Itga11/Bcl2/Col6a1/Col6a2/Col4a1/Col4a2/Itga9/Fn1/Thbs3/Shc2/Prkcg/Lamb3/Col6a6/Col4a4/Col4a3
mmu04926	Relaxin signaling pathway	18	18/339	129/8949	1.56E-06	Gng12/Adcy2/Tgfbr1/Creb3l2/Col1a1/Col1a2/Adcy7/Tgfb1/Col4a1/Col4a2/Gngt2/Mmp2/Gng8/Shc2/Fos/Ednrb/Col4a4/Col4a3
mmu05414	Dilated cardiomyopathy	15	15/339	94/8949	2.13E-06	Itga6/Itgb4/Adcy2/Itgb3/Itgb6/Tgfb3/Adcy7/Itga11/Tgfb1/Itga9/Tgfb2/Cacna2d2/Tpm4/Cacng4/Myh7
mmu05410	Hypertrophic cardiomyopathy	14	14/339	91/8949	7.26E-06	Itga6/Itgb4/Itgb3/Itgb6/Tgfb3/Itga11/Tgfb1/Ace/Itga9/Tgfb2/Cacna2d2/Tpm4/Cacng4/Myh7
mmu05146	Amoebiasis	15	15/339	107/8949	1.11E-05	Col1a1/Tgfb3/Col1a2/Cd14/Tgfb1/Col4a1/Col4a2/Tgfb2/Fn1/Tlr2/Prkcg/Lamb3/Col4a4/Casp3/Col4a3

### Screening of key circRNAs involved in cardiac hypertrophy and cardiac fibrosis

**FIGURE 7:**
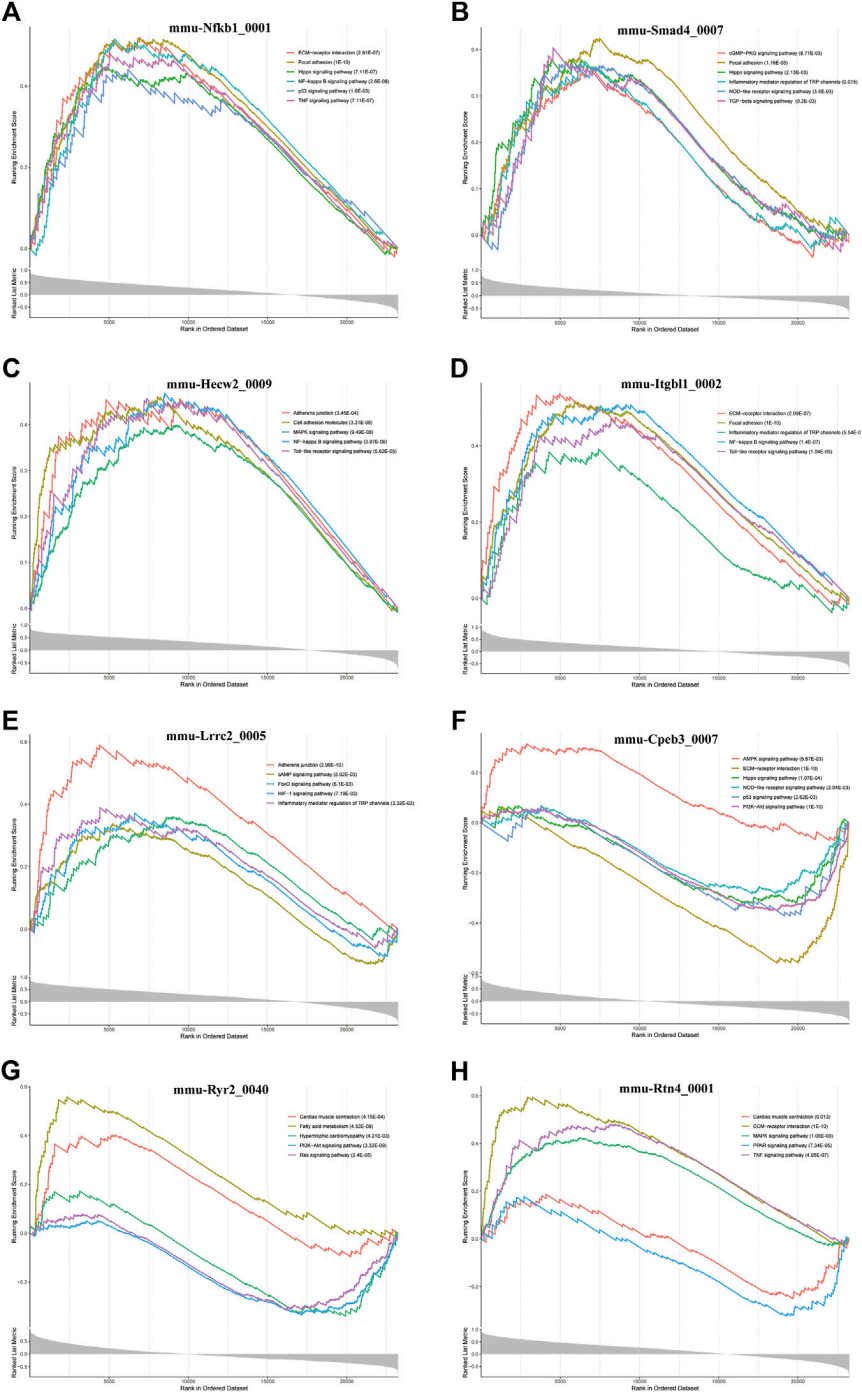
GSEA analysis of some key circRNAs. The pathways related to **(A)** mmu-Nfkb1_0001, **(B)** mmu-Smad4_0007, **(C)** In the pathways related mmu-Hecw2_0009, **(D)** mmu-Itgbl1_0002, **(E)** mmu-Lrrc2_0005, **(F)** mmu-Cpeb3_0007, **(G)** mmu-Ryr2_0040, and **(H)** mmu-Rtn4_0001.

Furthermore, we conducted GSEA of DE-circRNA-related coexpressed DE-mRNAs. We focused on circRNAs that involved in cardiac hypertrophy and cardiac fibrosis pathways and found that these circRNAs included mmu-Nfkb1_0001, mmu-Smad4_0007, mmu-Hecw2_0009, mmu-Itgbl1_0002, mmu-Lrrc2_0005, mmu-Cpeb3_0007, mmu-Ryr2_0040, and mmu-Rtn4_0001. Figure 7 demonstrates the pathways most associated with these circRNAs. Among them, mmu-Nfkb1_0001 was enriched in ECM-receptor interaction, focal adhesion, the Hippo signaling pathway, the NF-kappa B signaling pathway, the p53 signaling pathway and the TNF signaling pathway (Figure 7A). In the pathways related to mmu-Smad4_0007, the cGMP-PKG signaling pathway, inflammatory mediator regulation of TRP channels, the NOD-like receptor signaling pathway, and the TGF-beta signaling pathway were significantly activated (Figure 7B). Regarding the pathway associated with mmu-Cpeb3_0007, the AMPK signaling pathway was significantly activated, and ECM-receptor interactions and the Hippo, NOD-like receptor, p53 and PI3K-Akt signaling pathways were significantly inhibited (Figure 7F). In addition, mmu-Ryr2_0040 is associated with myocardial metabolism and cardiac function. For example, cardiac muscle contraction and fatty acid metabolism were significantly activated, and hypertrophic cardiac cardiomyopathy and the Ras signaling pathway were significantly inhibited (Figure 7G). Several other circRNA-related pathways, including the Toll-like receptor signaling pathway, HIF-1 signaling pathway and MAPK signaling pathway, were also activated or inhibited to varying degrees (Figures 7C–E,H). Notably, all alterations in these pathways are closely related to the inflammatory response, cardiac fibrosis process and cardiac function.

### Expressional and functional validations of key circRNAs

**FIGURE 8:**
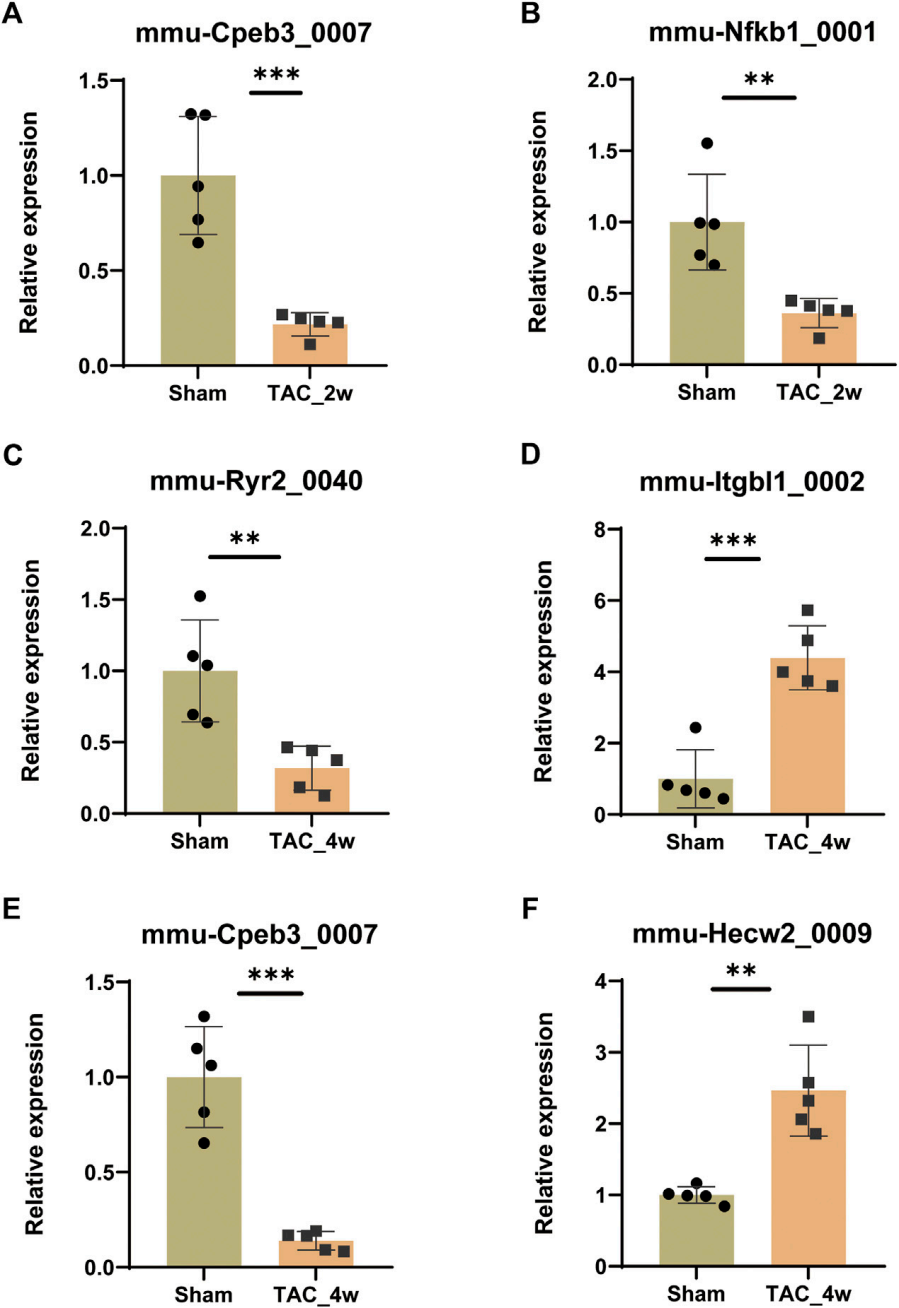
Validation of circRNA expression. The expression of **(A)**mmu-Cpeb3_0007, **(B)**mmu-Nfkb1_0001, **(C)**mmu-Cpeb3_0007, **(D)**mmu-Ryr2_0040, **(E)** mmu-Hecw2_0009 and **(F)** mmu-Itgbl1_0002 in the TAC_2w/TAC_4w group compared with the sham group. Results are presented as means ± standard error of the mean; ∗ indicates *p* < 0.05, ∗∗*p* < 0.01, n = 5.

To validate the expression of key DE circRNAs, we randomly selected 6 circRNAs with different expression trends from the 8 circRNA candidates screened by GSEA in the previous step. The qPCR results showed that the expression of mmu-Cpeb3_0007 and mmu-Nfkb1_0001 was downregulated in the TAC_2w group (Figures 8A,B), the expression of mmu-Ryr2_0040 and mmu-Cpeb3_0007 was downregulated in the TAC_4w group (Figures 8C,E), and the expression of mmu-Hecw2_0009 and mmu-Itgbl1_0002 was upregulated in the TAC4w group compared with the sham group (Figures 8D,F). And the circRNAs backspliced junction was further validated with sanger sequencing (Supplementary Figures S7). These results validated the reproducibility of the sequencing data.

Furthermore, we isolated cardiomyocytes and cardiac fibroblasts from neonatal mouse myocardium, and performed qRT–PCR to detect the expression of key circRNAs in these 2 cells. We found that mmu-Hecw2_0009, mmu-Nfkb1_0001, mmu-Cpeb3_0007 and mmu-Ryr2_0040 are mainly expressed in cardiomyocytes, mmu-Itgbl1_0002 is mainly expressed in cardiac fibroblasts (Supplementary Figures S6).

**FIGURE9:**
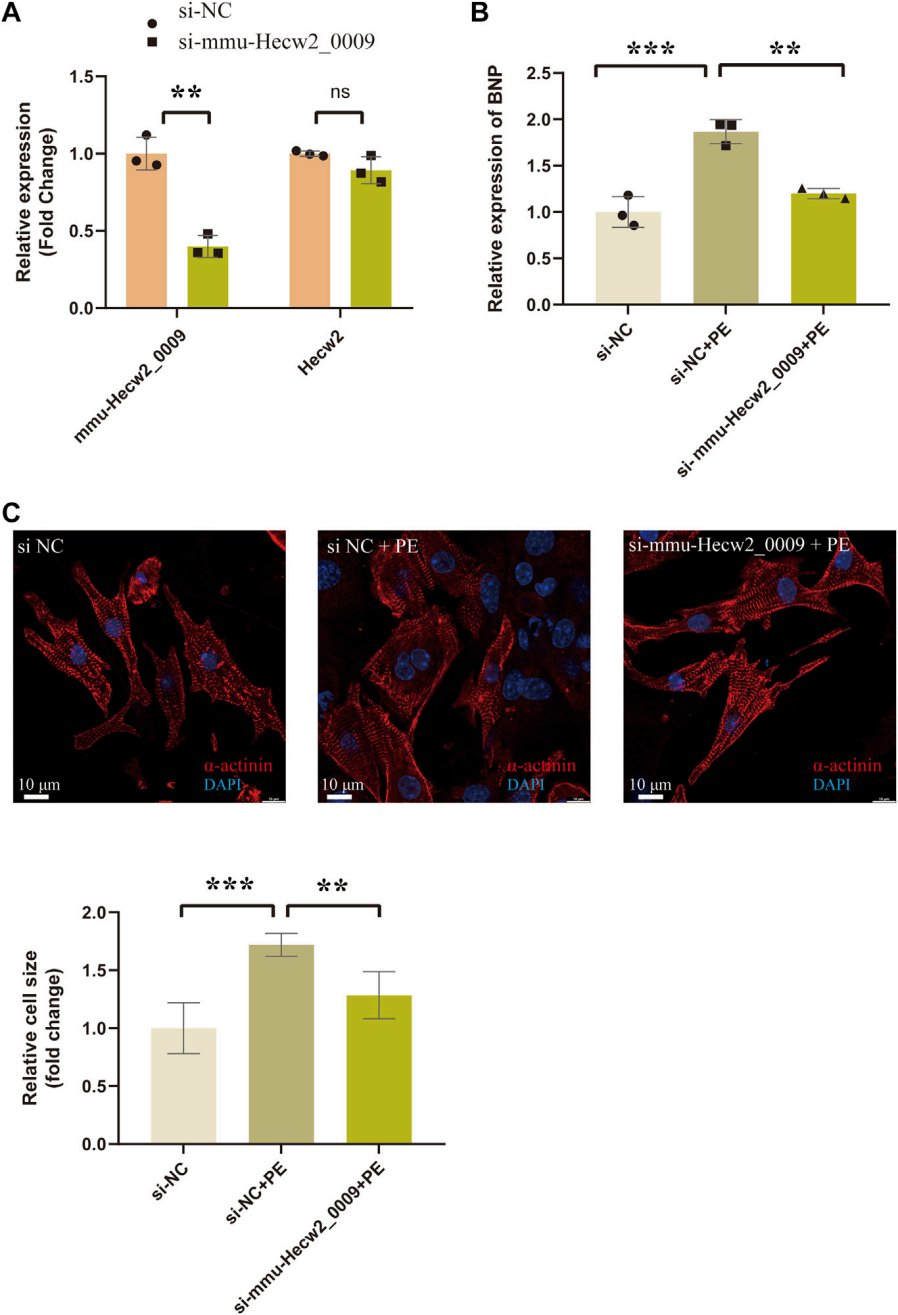
Knock-down of mmu-Hecw2_0009 reduced phenylephrine‐induced cardiomyocyte hypertrophy *in vitro*. **(A)** Expression of mmu-Hecw2_0009 and Hecw2 in NMCMs after si-mmu-Hecw2_0009 transfection (∗∗indicates *p* < 0.01, ∗∗∗*p* < 0.001,n = 3). **(B)**Morphologies of NMCMs with knok-down of mmu-Hecw2_0009 by α-actinin staining (α-actinin (red) and nuclei DAPI (blue)). **(C)**Expression of BNP mRNA in NMCMs with knok-down of mmu-Hecw2_0009 by RT-qPCR. (∗∗indicates *p* < 0.01, ∗∗∗*p* < 0.001, *n* = 3).

Among these circRNAs in Figure 8, mmu-Hecw2_0009 was increased in TAC_4w and abundantly expressed in cardiomyocytes. Therefore, we synthesized siRNA targeting mmu-Hecw2_0009 to explore its function in cardiomyocyte hypertrophy. The results showed that the expression of cardiac hypertrophy marker gene BNP was increased in si-NC+PE group compared with si-NC group, and treatment with si-mmu-Hecw2_0009 decreased mRNA expression of BNP in si-mmu-Hecw2_0009+PE group compared with si-NC+PE group (Figures 9A,B). The immunofluorescence microscopy experiments showed that the size of cardiomyocytes was significantly increased in si-NC+PE group compared with si-NC group, and knock-down of mmu-Hecw2_0009 reduced phenylephrine‐induced cardiomyocyte hypertrophy *in vitro* (Figure 9C).

### ceRNA network construction

**FIGURE 10:**
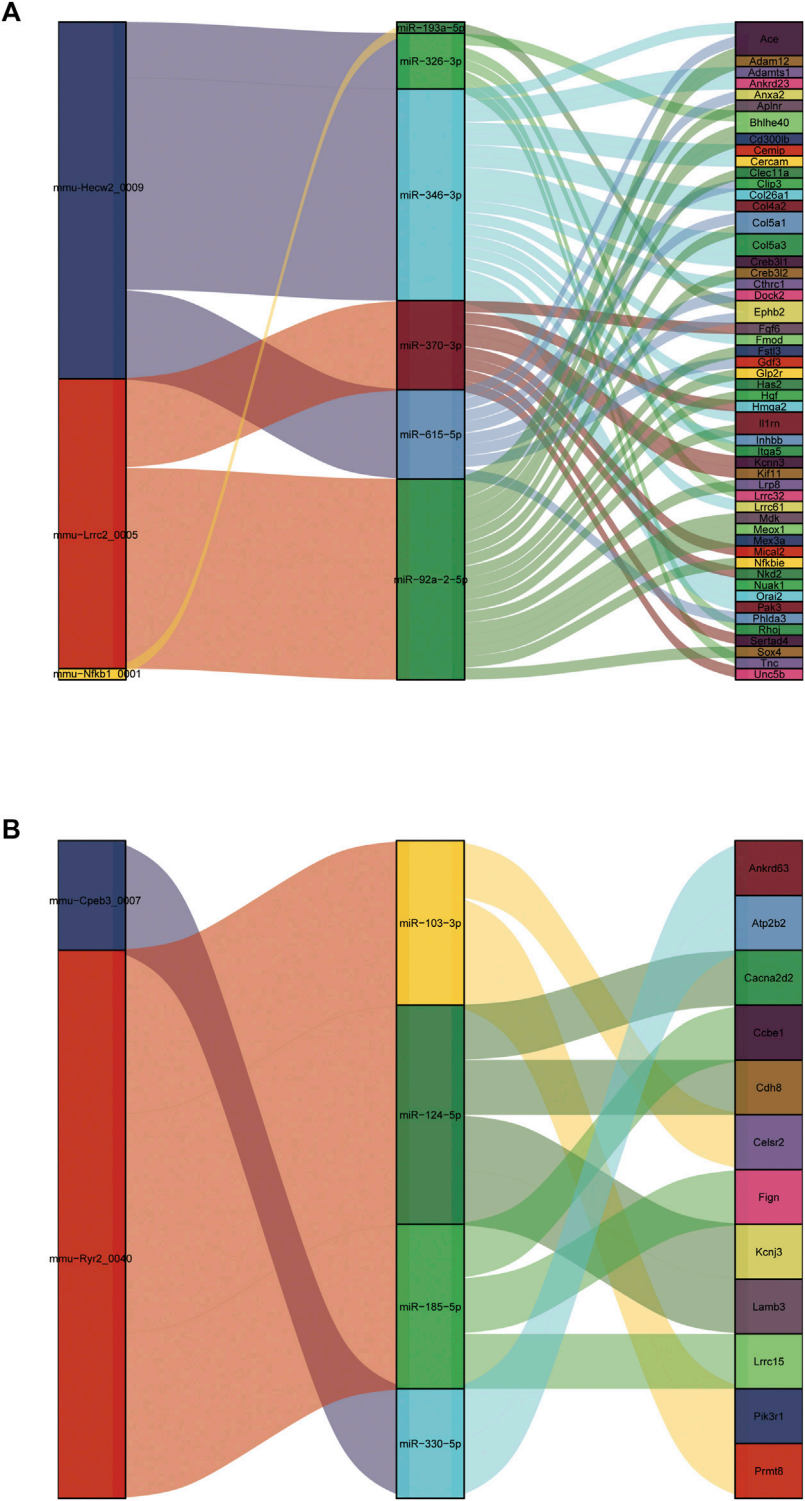
Construction of the circRNA-based ceRNA network. **(A)** The ceRNA network of up circRNA-miRNA-up mRNA pattern. **(B)** The ceRNA network of down circRNA-miRNA-down mRNA pattern.

It is well known that miRNAs negatively regulate gene expression by binding to mRNA posttranscriptionally. CircRNAs can act as competing endogenous RNAs (ceRNAs) for miRNA and counteract the repressive activity of miRNA (Salmena et al., 2011). Based on the ceRNA theory, we first integrated the DE circRNAs and DE mRNAs with their targeted miRNAs. Then, RNAhybrid and miRanda were used to predict circRNA-miRNA pairs, and TargetScan, miRDB, miRTarBase and miRWalk were used to predict miRNA–mRNA pairs. Finally, we used DE circRNAs as decoys, miRNAs as centers, and DE mRNAs as targets to construct a possible circRNA-miRNA–mRNA regulatory network. A total of five circRNAs, 10 miRNAs and 64 mRNAs were involved in the ceRNA regulatory networks (Figure 10). We divided ceRNA regulatory networks into two groups based on their gene expression patterns. Specifically, Figure 10A presents up circRNA-miRNA-up mRNA, and Figure 10B presents down circRNA-miRNA-down mRNA. In the ceRNA networks, we observed several valuable axes, including mmu-Hecw2_0009---miR-346-3p---Col4a2/Col5a3/Cthrc1/Fmod/Inhbb/Orai2/Cemip, mmu-Lrrc2_0005---miR-92a-2-5p---Sox4/Meox1/Nfkbie/Mdk/Il1rn/Col5a1/Creb3l2, mmu-Cpeb3_0007---miR-92a-2-5p---Cacna2d2/Cdh8/Kcnj3, mmu-Ryr2_0040---miR-185-5p---Ccbe1/Lrrc15, and mmu-Ryr2_0040---miR-103-3p---Celsr2/Prmt8.

## Discussion

In our study, we characterized the phenotypic, cardiac function and pathological changes during cardiac hypertrophy and fibrosis induced by pressure overload. Two weeks after TAC, cardiac compensatory hypertrophy occurs. In the early hypertrophic stage, the cardiac function of mice did not change significantly. Four weeks after TAC, heart weight, cardiomyocyte hypertrophy, and cardiac fibrosis further increased. Additionally, in this stage, the cardiac function of the mice deteriorated. The identification of these key features facilitates the subsequent comprehensive analysis of molecular expression patterns in combination with these pathological features.

Previous studies have suggested that cellular metabolism, immune responses, translation regulation and epigenetic modification regulate cardiac hypertrophy (Nakamura and Sadoshima, 2018). A number of factors, such as adrenergic, GPCRs, endothelin (ET-1) receptors and angiotensin, are all key regulators in cardiac hypertrophy (Everett et al., 1994; Rockman et al., 2002). Recent studies have indicated that circRNA and circRNA-based ceRNA network regulation have emerged as an indispensable modality for posttranscriptional regulation of mRNA in cardiac hypertrophy. Wang and his colleagues found that circRNA HRCR acts as an endogenous sponge of miR-223 to regulate ARC expression in cardiac hypertrophy (Wang et al., 2016). CircSlc8a1, interacting endogenously with miR-133a in cardiomyocytes, may serve as a promising therapeutic target for cardiac hypertrophy (Lim et al., 2019). Recently, a new algorithm, CIRIquant, was used to sensitively discover unknown circRNAs based on whole transcriptome data. In our study, identification and quantification of circular RNAs were accurately performed by CIRIquant. Furthermore, by analyzing the function of circular RNAs in cardiac hypertrophy and cardiac fibrosis, we found that these circRNAs potentially regulate the oxidative stress response, lipid metabolism, inflammatory response, ECM-receptor interaction and focal adhesion during cardiac hypertrophy. Integrating DE circRNAs and the function of coexpressed circRNA-mRNA, we focused on some key circRNAs closely associated with inflammation, cardiac metabolism and cardiac fibrosis, including mmu-Nfkb1_0001, mmu-Smad4_0007, mmu-Hecw2_0009, mmu-Itgbl1_0002, mmu-Lrrc2_0005, mmu-Cpeb3_0007, mmu-Ryr2_0040, and mmu-Rtn4_0001.

In the circRNA-based ceRNA regulatory network, some miRNAs and mRNAs directly regulate cardiac hypertrophy or cardiac fibrosis. Previous research has shown that miR-103-3p is negatively linked to cardiomyocyte fate (Dhingra et al., 2015; Wang et al., 2015). Zhang *et al.* indicated that overexpression of cytosolic long noncoding RNA cytb protects against pressure-overload-induced heart failure by sponging microRNA-103-3p (Zhang et al., 2022). In our study, we found that the mmu-Ryr2_0040---miR-103-3p---Celsr2/Prmt8 axis may be a novel target in cardiac hypertrophy. Lu’s study reported that Meox1 accelerated myocardial hypertrophic decompensation through Gata4. Our identification of the mmu-Lrrc2_0005---miR-92a-2-5p---Meox1 axis may serve as a novel mechanism by which Meox regulates cardiac hypertrophy. Besides, Col4a2, a basal lamina protein associated with interstitial fibrosis, is predicted to regulate fibrosis in hypertrophic cardiomyopathy (Maron et al., 2021). Col5a3 is also a fibrotic target gene (Dey et al., 2020). Interestingly, mmu-Hecw2_0009 was upregulated after TAC and mmu-Hecw2_0009 may regulate Col4a2 and Col5a3 by inhibiting the activity of miR-346-3p. Li *et al.* demonstrated that Cthrc1 is a new regulator of liver fibrosis by modulating TGF-β signaling (Li et al., 2019). It is worth exploring whether Cthrc1 regulates cardiac fibrosis and whether its mechanism is mediated by the mmu-Hecw2_0009-miR-346-3p-Cthrc1 axis. In addition to the above, Inhbb is implicated in the pathogenesis of renal fibrosis by activating the surrounding fibroblasts (Sun et al., 2022). It may also be worth exploring whether the mmu-Hecw2_0009---miR-346-3p---Inhbb axis plays a role in cardiac fibrosis.

Three studies have recently analyzed the ceRNA network in cardiac hypertrophy/cardiac fibrosis/heart failure (Gu et al., 2020; Chen et al., 2022; Li et al., 2022). Li *et al.* demonstrated distinct pathological features in the three stages of heart failure and constructed a ceRNA regulatory network in cardiac remodeling and dysfunction (Li et al., 2022). In their study, arraystar microarrays were used to identify circRNAs. However, most of the circRNA sequences obtained by microarrays are known, which results in limited circRNA information. Moreover, two samples from each group were analyzed in the sequencing data, which raises concerns that few biological replicates may cause some unexpected bias. In our study, we had more replicates per group (n = 5), and whole transcriptome RNA-seq was used. Chen *et al.* used data from human iPSC-derived cardiomyocytes from public databases (Aggarwal et al., 2014) and established a circRNA-mediated ceRNA regulatory network in cardiac hypertrophy (Chen et al., 2022), which may not be representative of specific physiopathological features during cardiac hypertrophy. Gu et al. determined the circRNA expression profile and conducted constructed a ceRNA network in TGF-β1-treated mouse cardiac fibroblasts. However, the occurrence of cardiac fibrosis *in vivo* is complex and multifactorial, and the activation of cardiac fibroblasts induced by TGF-β1 *in vitro* cannot completely mimic the cardiac fibrosis *in vivo*. In summary, previous circ-based ceRNA studies in cardiac hypertrophy and cardiac fibrosis have some limitations, such as insufficient sample size and outdated circular RNA annotation databases. In addition, some studies have conducted functional studies of circRNAs in TAC models. Wang et al. reported that circ-SIRT1 inhibits cardiac hypertrophy via activating SIRT1 to promote autophagy. Specifically, in their study, ceRNA network play an important role in pathogenesis of cardiac hypertrophy. Circ-SIRT1 bound with miR-3681-3p and miR-5195-3p to regulate SIRT1 expression post-transcriptionally (Wang et al., 2021). In another study, Wu et al. indicated that circ Yap is a critical regulator in cardiac fibrosis in the pressure overload mouse model (Wu et al., 2021). These studies deeply explored the function and mechanism of circRNA in cardiac hypertrophy and fibrosis by constructing a TAC model, which is need for further studies. However, the above-mentioned study did not show the circRNAs map so that we could not obtain more information about the function of other unknown aberrant circRNAs in TAC-induced cardiac dysfunction.

Therefore, in our study, we have explored the expression and possible functional mechanisms of some key circRNAs in dysregulated process of heart in our study mainly based on established bioinformatics methods and some basic experiments. Then, we constructed a ceRNA regulatory network to shed new light on the process of cardiac hypertrophy and cardiac fibrosis. Our results may provide promising targets for the treatment of cardiac hypertrophy and cardiac fibrosis. However, in-depth functional verification experiments and mechanism research are also necessary in the future.

## Data Availability

The datasets presented in this study can be found in online repositories. The names of the repository/repositories and accession number(s) can be found in the article/Supplementary Material.
