# Supplementary figures and images for: Identification of circular RNAs in cardiac hypertrophy and cardiac fibrosis
Source: Front Pharmacol. 2022 Aug 8;13:940768. doi: 10.3389/fphar.2022.940768 (PMC9393479; doi:10.3389/fphar.2022.940768)

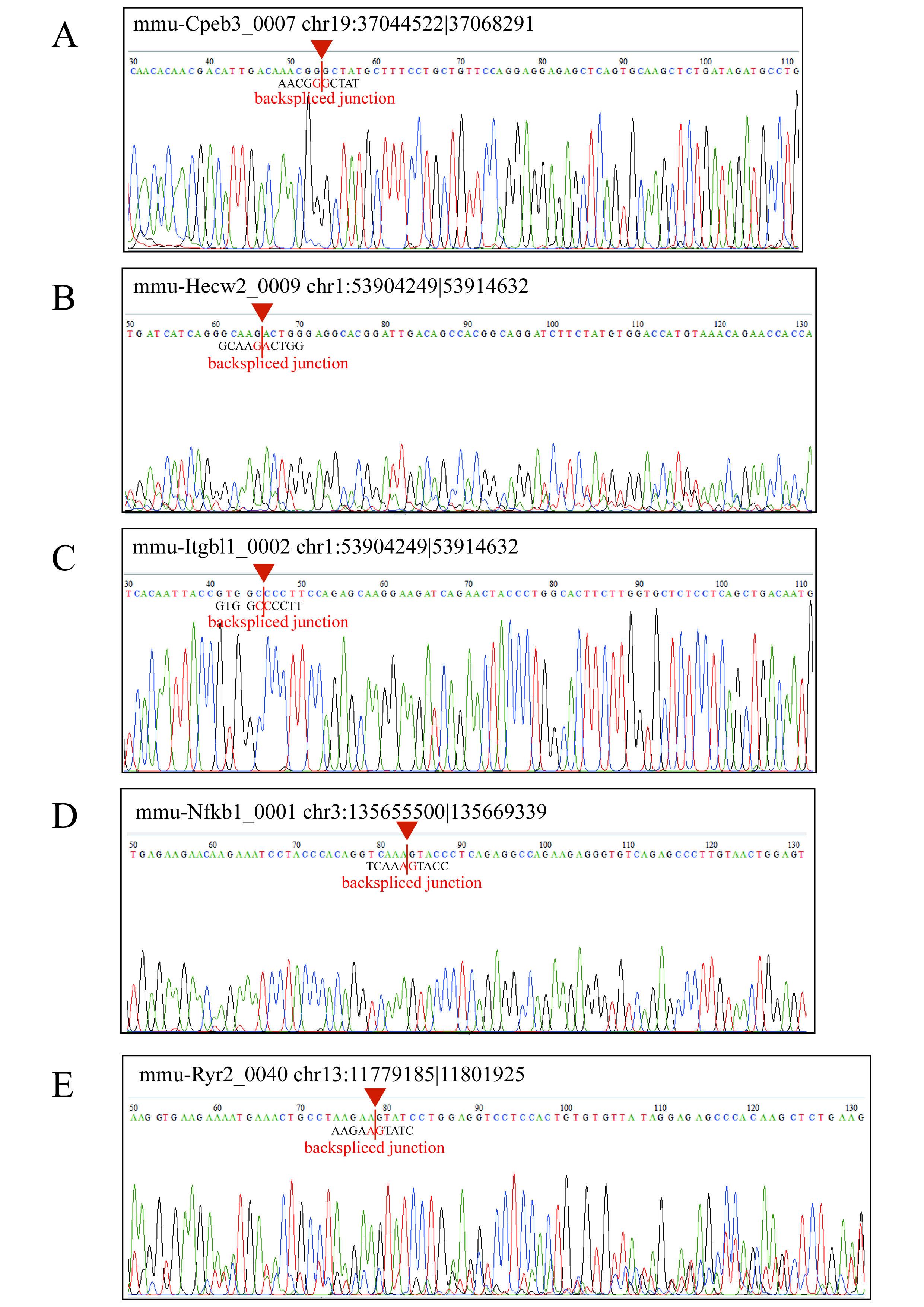

Supplement: Supplementary file 3 [file Image6.TIF]

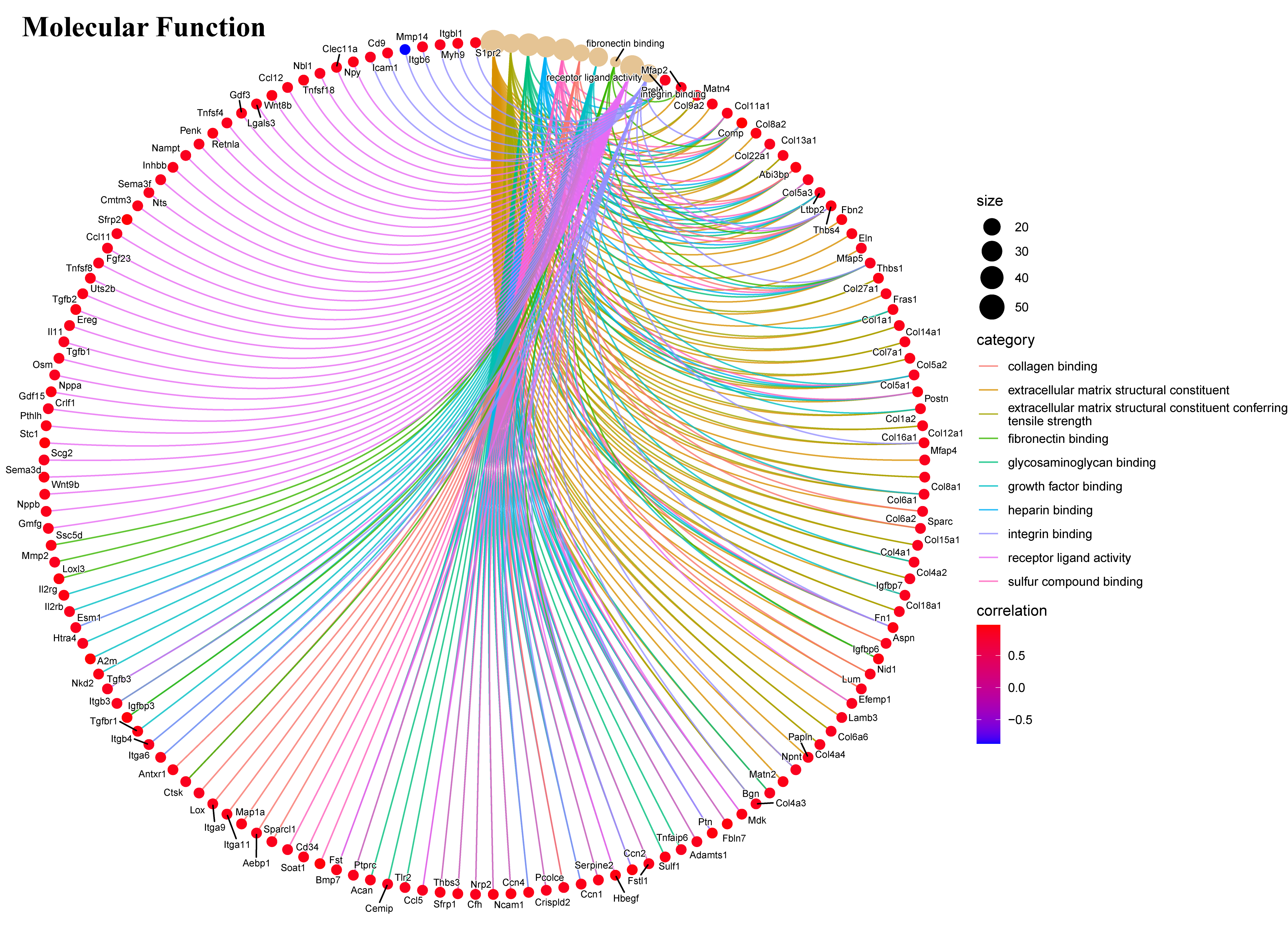

Supplement: Supplementary file 4 [file Image3.TIF]

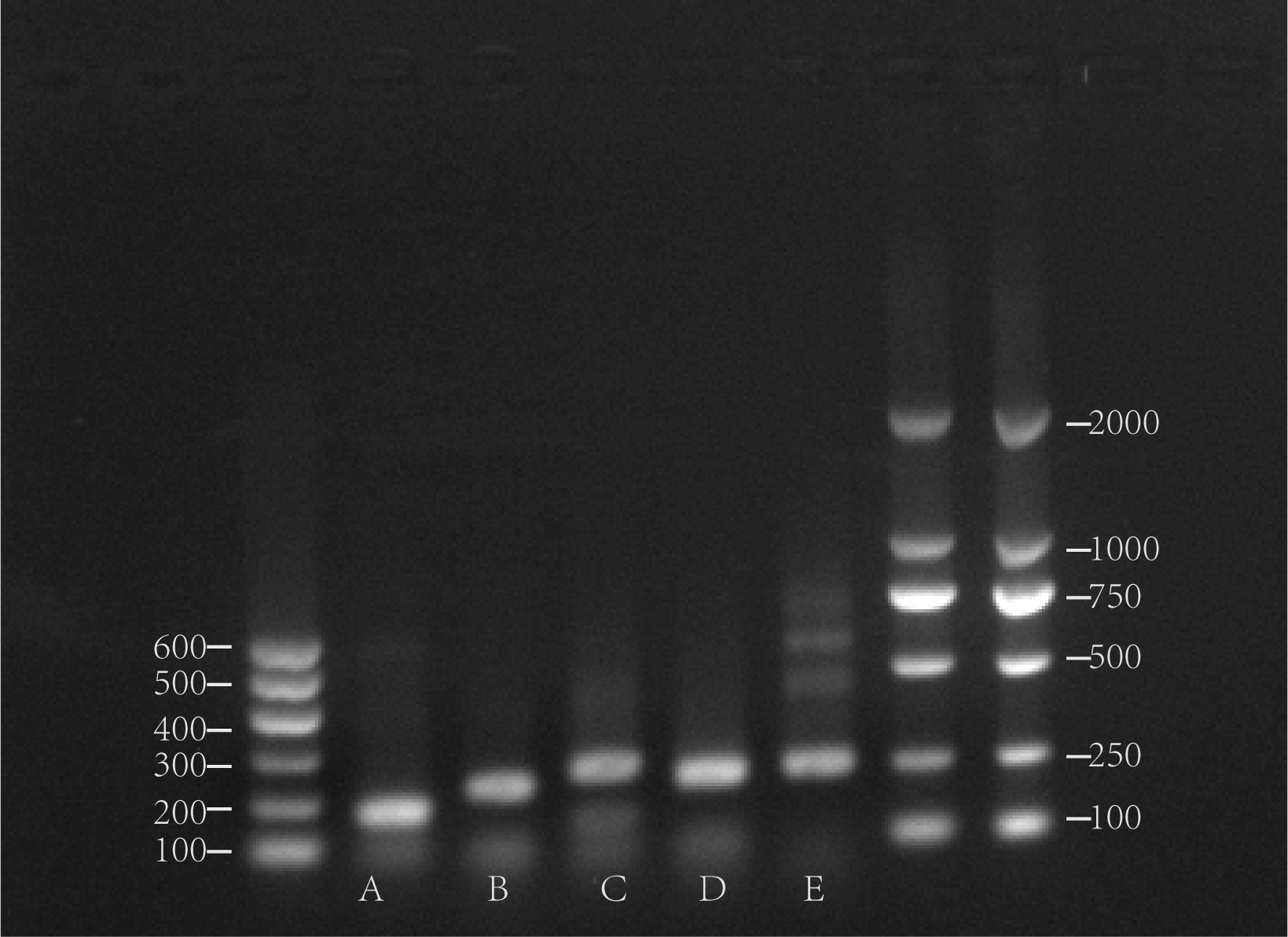

Supplement: Supplementary file 5 [file Image4.TIF]

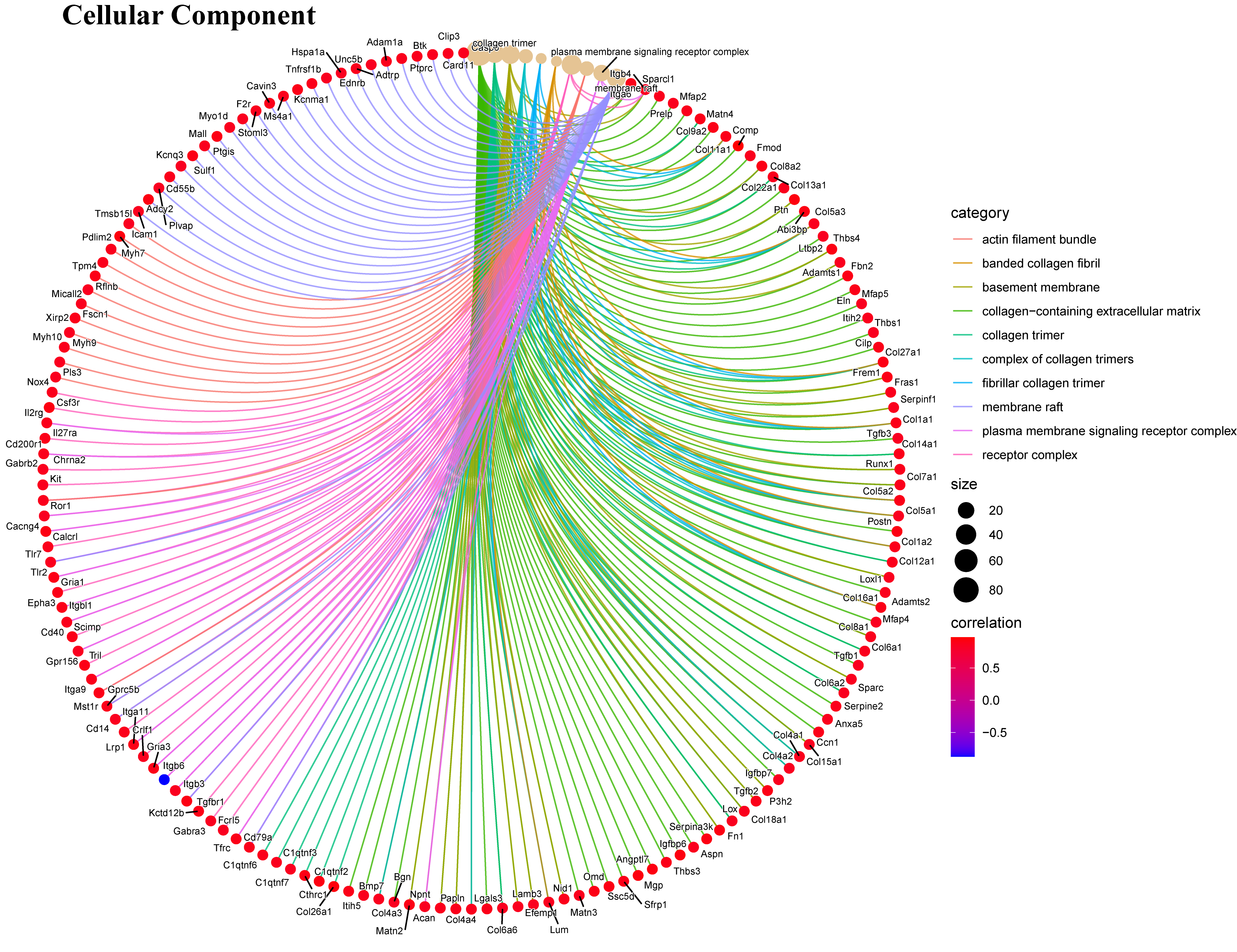

Supplement: Supplementary file 6 [file Image2.TIF]

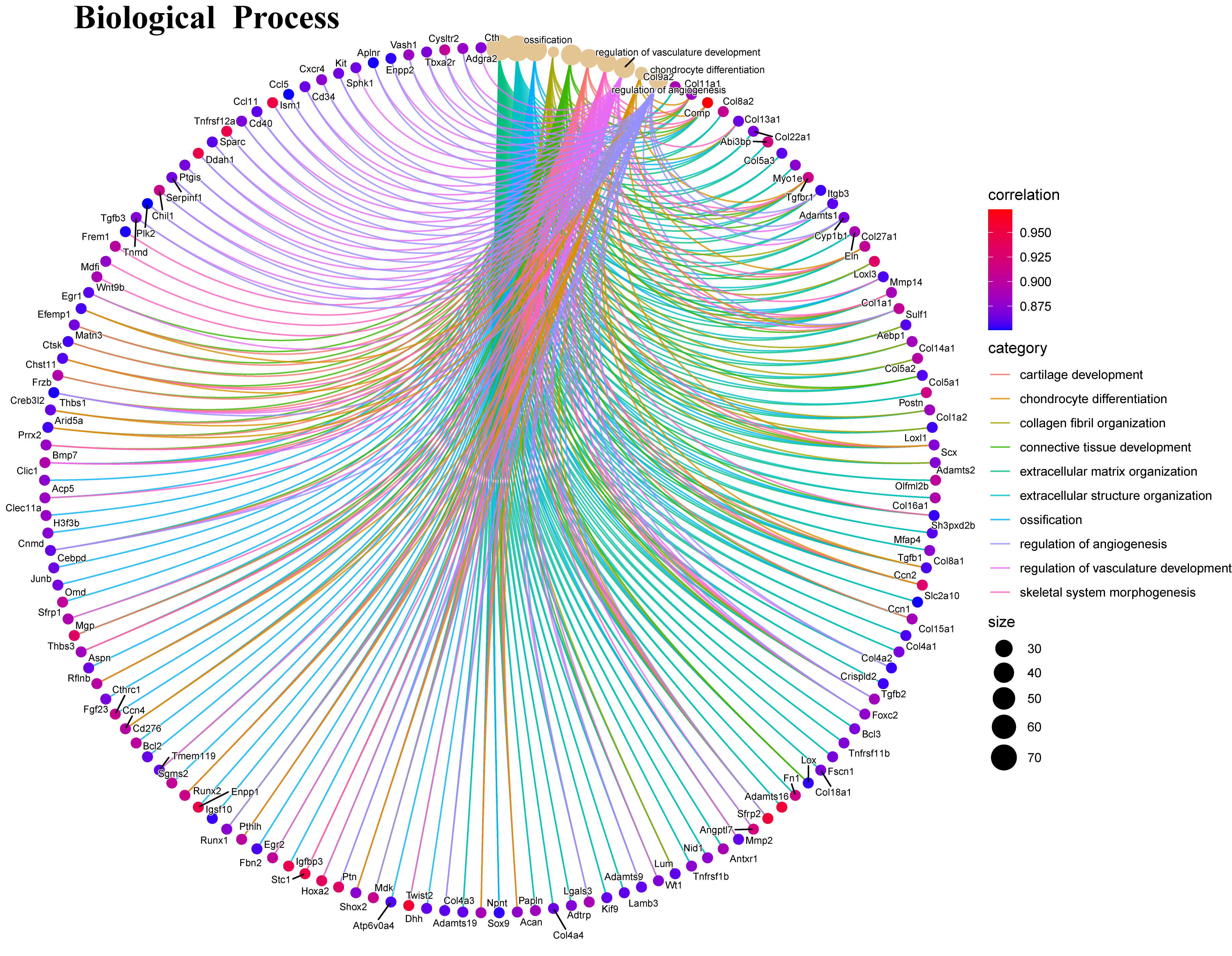

Supplement: Supplementary file 7 [file Image1.TIF]

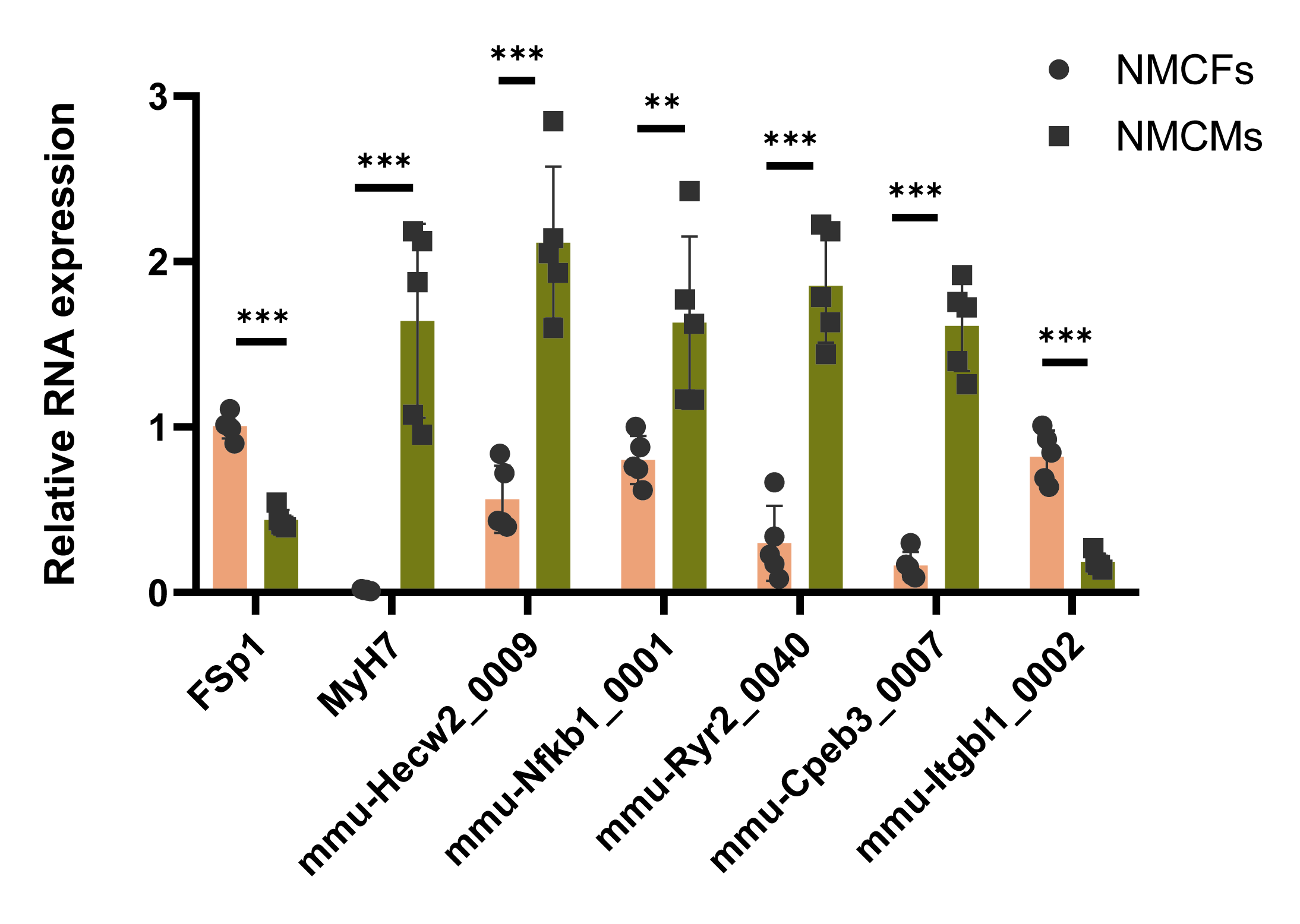

Supplement: Supplementary file 11 [file Image5.TIF]
