# Supplementary material for: Identification of circular RNAs in cardiac hypertrophy and cardiac fibrosis
Source: Front Pharmacol. 2022 Aug 8;13:940768. doi: 10.3389/fphar.2022.940768 (PMC9393479; doi:10.3389/fphar.2022.940768)
Supplement: Supplementary file 8 [file Table5.DOCX]

| Supplemental Table 1. Sequences of qRT-PCR primers | |
| --- | --- |
| Genes | Primer sequence (5′-3′) |
| mmu-Itgbl1_0002 | F: GTACTGACAAGCTTCCCCAGA |
|  | R: GTTCAGGTCATGGGCAGTGTA |
| mmu-Hecw2_0009 | F: GCGCTATCAGCATAACAGGG |
| mmu-Ryr2_0040 | R: CTGCGGATGCTCTGATAACG  F: GCAGATGTCGCTTGAAACCC  R: GGACCTTGTGGTTTCTCCCA |
| mmu-Cpeb3_0007 | F: GTTGCGTTCTCCAATCAGCAG |
|  | R: TACAGTTTCCCATCCTCCTCCA |
| mmu-Nfkb1_0001 | F: AGCAACCAAAACAGAGGGGAT |
| BNP  Hecw2 | R: AATCTTTGGGCTGGAGTGAGC  F: GGAGGAAATGGCCCAGAGAC  R: CAGTGCGTTACAGCCCAAAC  F: AGAGCCCACACTTGTTTTAACC  R: TCCAAAGGTACTGGTCTCTTCAA |
| mmu-β-actin | F: GTGACGTTGACATCCGTAAAGA  R: GCCGGACTCATCGTACTCC |
